# Screening of Secondary Metabolites Produced by *Nigrospora sphaerica* Associated with the Invasive Weed *Cenchrus ciliaris* Reveals Two New Structurally Related Compounds

**DOI:** 10.3390/molecules29020438

**Published:** 2024-01-16

**Authors:** Maria Michela Salvatore, Maria Teresa Russo, Susan Meyer, Angela Tuzi, Marina Della Greca, Marco Masi, Anna Andolfi

**Affiliations:** 1 Department of Chemical Sciences, University of Naples Federico II, 80126 Naples, Italy; mariamichela.salvatore@unina.it (M.M.S.); mariateresa.russo2@unina.it (M.T.R.); angela.tuzi@unina.it (A.T.); dellagre@unina.it (M.D.G.); andolfi@unina.it (A.A.); 2 Department of Geosciences, Southern Utah University, Cedar City, UT 84721, USA; susanmeyer@suu.edu; 3 BAT Center-Interuniversity Center for Studies on Bioinspired Agro-Environmental Technology, University of Naples Federico II, 80055 Portici, Italy

**Keywords:** specialized metabolites, buffelgrass, phytopathogen, bioherbicide, metabolomics

## Abstract

In the search for new alternative biocontrol strategies, phytopathogenic fungi could represent a new frontier for weed management. In this respect, as part of our ongoing work aiming at using fungal pathogens as an alternative to common herbicides, the foliar pathogen *Nigrospora sphaerica* has been evaluated to control buffelgrass (*Cenchrus ciliaris*). In particular, in this work, the isolation and structural elucidation of two new biosynthetically related metabolites, named nigrosphaeritriol (3-(hydroxymethyl)-2-methylpentane-1,4-diol) and nigrosphaerilactol (3-(1-hydroxyethyl)-4-methyltetrahydrofuran-2-ol), from the phytotoxic culture filtrate extract were described, along with the identification of several known metabolites. Moreover, the absolute stereochemistry of (3*R*,4*S*,5*S*)-nigrosphaerilactone, previously reported as (3*S*,4*R*,5*R*)-4-hydroxymethyl-3,5-dimethyldihydro-2-furanone, was determined for the first time by X-ray diffraction analysis. Considering their structural relationship, the determination of the absolute stereochemistry of nigrosphaerilactone allowed us to hypothesize the absolute stereochemistry of nigrosphaeritriol and nigrosphaerilactol.

## 1. Introduction

Natural products and microorganisms have been recognized as valuable biopesticides worldwide [1,2,3]. However, synthetic pesticides represent the current primary weapons to control pests found in agricultural crops and, due to their long-term use, they are responsible for several adverse effects including carcinogenicity, teratogenicity, and high and acute residual toxicity [4,5,6]. Their intensive use and relatively long environmental persistence have led to the contamination of wastewater and soil, and the presence of residues in food is becoming an important issue for consumers [1,7,8]. Hence, considering the hazardous effects of synthetic pesticides, there is an urgent need for alternative strategies in managing the agricultural pests. Microbial technologies could play a crucial role in reducing reliance on synthetic pesticides [9,10,11]. For the near future, it is expected that biopesticides will become increasingly important as crop protection products promoting sustainable agriculture related to their reduced impact on human health and the environment, both in individual applications and within integrated pest management (IPM) [12]. This latter approach is particularly interesting because its application involves the use of a combination of control methods, taking into account the specific characteristics of the invasive species and the local ecosystem [13].

Herbicidal products are a large sector of the pesticide market because weeds create serious constraints in agricultural production. Invasive weed genera, including *Cenchrus*, *Cuscuta*, *Hydrilla*, *Lantana*, *Parthenium*, and *Salvinia* are a potential threat to biodiversity and can also impact crops [14,15,16,17]. In particular, controlling the spread of *Cenchrus ciliaris*, a warm-season grass commonly known as buffelgrass, has become one of the major challenges. In fact, in places like the Sonoran Desert in the southwestern United States, buffelgrass can outcompete native vegetation, and due to its high flammability, it increases the frequency and intensity of wildfires, posing a threat to both human communities and native flora and fauna [18,19]. The available strategies for buffelgrass management include manual removal and controlled burns, but most involve the problematic use of synthetic herbicides [20]. The utilization of microbial bioproducts could be an efficient alternative to buffelgrass chemical control. In fact, microorganisms associated with buffelgrass likely developed strategies to contend with neighboring plant competitors so they can be used for the development of eco-friendly herbicidal formulations with new mechanisms of action [21].

As part of our ongoing research aimed at using the phytotoxins produced by buffelgrass foliar pathogens for the development of a bioherbicide that could be used in IPM practices, three foliar pathogens isolated from diseased *C. ciliaris* leaves, namely *Cochliobolus australiensis*, *Pyricularia grisea*, and *Nigrospora sphaerica*, have been evaluated as potential sources of phytotoxins for buffelgrass control in its North American range. In our previous papers, *P. grisea* and *C. australiensis* have been reported as important sources of secondary metabolites that could potentially be used as natural herbicides against this weed [22,23,24].

Following our demonstration of a strong phytotoxic activity against buffelgrass seeds of the culture filtrate extract of a strain of *N. sphaerica* isolated from buffelgrass (*C. ciliaris*) leaves, in this work, a secondary metabolite screening of culture filtrate and mycelial extracts was conducted. In particular, the present paper reports the structural elucidation of a new triol named nigrosphaeritriol (**1**) and a new lactol named nigrosphaerilactol (**2**), together with the identification of several known compounds, i.e., nigrosphaerilactone (**3**), (*S*)-hydroxybutyrolactone (**4**), lupinlactone (**5**), nigrosporione A (**6**), 2,4-dihydroxy-6-methoxyacetophenone (**7**), tyrosol (**8**), 4-hydroxyphenilacetic acid (**9**), adenosine (**10**), uridine (**11**), ribo-hexonic acid 3-deoxy-γ-lactone (**12**), levoglucosan (**13**), 3-hydroxyisobutyric acid (**14**), and ergosta-7,22-dien-3-ol (**15**).

## 2. Results

### 2.1. Demonstration of Phytotoxicity in a Culture Filtrate Extract of Nigrospora sphaerica

**Figure 1:**
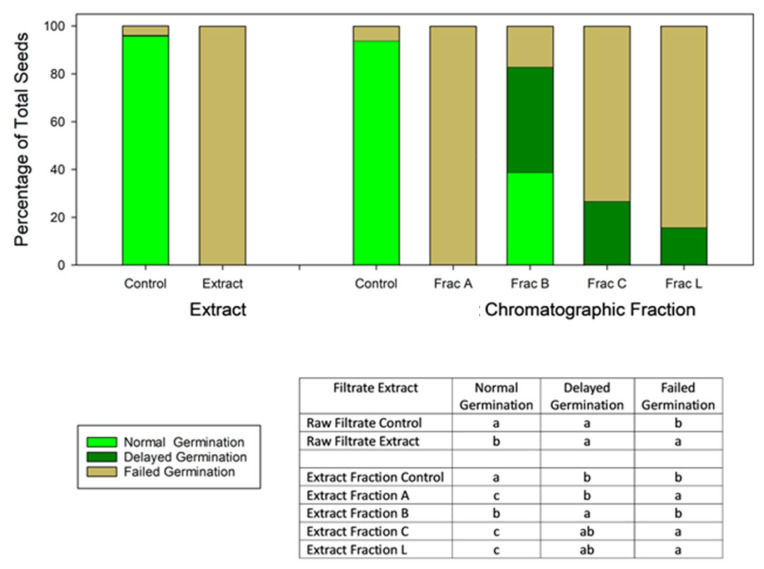
Results of *C. ciliaris* seedling bioassay tests with *N. sphaerica* raw filtrate extract and with each of four extract chromatographic fractions, showing normal, delayed, and failed germination percentages in each along with 2% DMSO controls. Means separations (Ryan–Einot–Gabriel–Welsch multiple range test; *p* < 0.05) in the inset panel are from analysis of variance for each variable performed separately on the raw extract and its control and on the four extract chromatic fractions and their common control.

The crude extract from the culture filtrate of *N. sphaerica* isolated from buffelgrass (*C. ciliaris*) showed no germination in a coleoptile and radicle elongation bioassay when tested at 2 mg mL^−1^ (Figure 1; see Materials and Methods). The untreated control in this bioassay germinated to >95% in <7 days. Subsequently, the crude extract was purified by combined chromatographic column (CC) and thin layer chromatography (TLC), obtaining ten homogeneous fractions. Only four of these fractions were obtained in sufficient quantity for bioassay and chemical characterization, and all proved to contain toxic substances that negatively impacted buffelgrass germination to at least some extent (Figure 1). Treatment with fraction A resulted in complete failure of germination, suppressing germination or resulting in aborted germination (coleorhiza exserted but no subsequent development of radicle or coleoptile) in all seeds, while fractions C and L were also highly toxic but resulted in a small fraction of seeds with delayed germination. Fraction B was much less toxic, with a failed germination percentage not significantly different from the control; however, its mild toxicity was evidenced by delayed germination in over half of the seeds that did eventually germinate.

### 2.2. Isolation and Characterization of Metabolites from Culture Filtrate Extract

**Figure 2:**
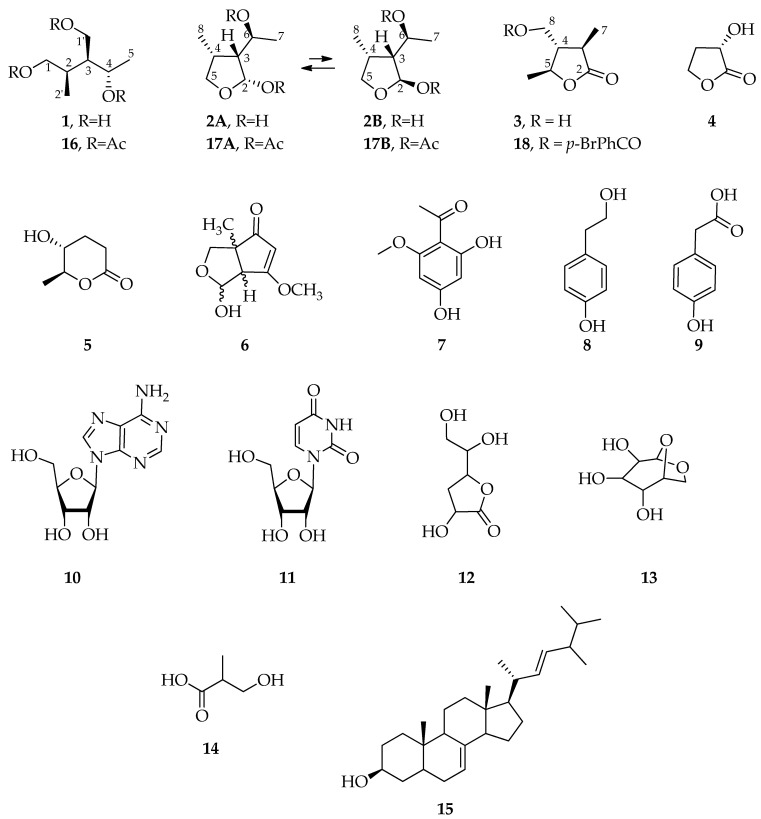
Chemical structures of (2*R*,3*S*,4*S*)-nigrosphaeritriol (**1**), (2*R*/2*S*, 3*R*,4*R*,6*S*)-nigrosphaerilactol (**2**), (3*R*,4*S*,5*S*)-nigrosphaerilactone (**3**), (*S*)-hydroxybutyrolactone (**4**), lupinlactone (**5**), nigrosporione A (**6**), 2,4-dihydroxy-6-methoxyacetophenone (**7**), tyrosol (**8**), 4-hydroxyphenilacetic acid (**9**), adenosine (**10**), uridine (**11**), ribo-hexonic acid 3-deoxy-γ-lactone (**12**), levoglucosan (**13**), 3-hydroxyisobutyric acid (**14**), ergosta-7,22-dien-3-ol (**15**), 1,1′,4-*O*,*O*′,*O*″-triacetylnigrosphaeritriol (**16**), 2,6-*O*,*O*′-diacetylnigrosphaerilactol (**17**), and 8-*O*-*p*-bromobenzoylnigrosphaerilactone (**18**).

The purification of active fractions led to the isolation of a new triol named nigrosphaeritriol (**1**) and a new lactol named nigrosphaerilactol (**2**), along with the identification of several known compounds (**3**–**15**) (Figure 2).

Nigrosphaeritriol (**1**) showed a molecular formula of C_7_H_16_O_3_, as deduced from the molecular ion cluster at *m*/*z* 149.1183 [M + H]^+^ (calcd for C_7_H_17_O_3_, 149.1178) in the high-resolution electrospray ionization mass spectrometry (HR-ESI-MS) spectrum (Figure S1) and no degrees of unsaturation (hydrogen deficiency index = 0). The analysis of its ^13^C NMR spectrum (Figure S2) revealed seven resonances belonging to three oxygenated and four aliphatic carbons (Table 1). In addition, the detailed investigation of its ^1^H NMR spectrum (Figure S3) showed the characteristic resonances of two methyl groups appearing at δ 0.98 (Me-2′) and 1.24 (Me-5) as doublets (*J* = 7.1 and 6.4 Hz, respectively), two methine protons appearing as two multiplets at δ 1.60 (H-3) and 2.05 (H-2), an oxygenated methine proton as a quintet (*J* = 6.4 Hz) at δ 3.90 (H-4), and two oxygenated methylene groups as a multiplet at δ 3.62 (H_2_-1′) and as two as two double doublets (*J* = 10.7 and 5.9 Hz) at δ 3.51 and 3.55 (*J* = 10.7 and 6.1 Hz) (H_2_-1), respectively. The COSY spectrum (Figure S4) evidenced the correlations of H_2_-1 with H-2, H-2 with H-3 and Me-2′, H-3 with H-4 and H_2_-1′, and H-4 with Me-5, while the seven carbons were assigned to the corresponding protons based on the correlations observed in the HSQC spectrum (Figure S8). In particular, the signals at δ 67.1, 66.2, 59.7, 48.7, 34.0, 20.1, and 12.3 were assigned to C-4, C-1, C-1′, C-3, C-2, C-5, and C-2′, respectively. The structure was supported by the long-range couplings observed in the HMBC spectrum (Figure S6, Table 1), and nigrosphaeritriol (**1**) was formulated as a new 3-(hydroxymethyl)-2-methylpentane-1,4-diol.

The EI mass spectrum at 70 eV of the trimethylsilyl derivative of nigrosphaeritriol shows a fragmentation pattern which confirms the attributed chemical structure (e.g., 291 *m*/*z* [M–TMS]^+^, 259 *m*/*z* [M–OTMS]^+^, 217 *m*/*z* [M–2TMS]^+^, and 73 *m*/*z* [TMS]^+^) (Figure S7).

The structure of compound **1** was confirmed by preparing ester derivatives by acetylation. The ^1^H NMR spectrum of derivative **16** (Figure S8, Table 1) differed from that of nigrosphaeritriol (**1**), recorded in the same conditions, for the presence of three singlets of the acetyl groups at *δ* 2.04, 2.05 and 2.7 and for typical downfield shift of H_2_-1 appearing as two double doublets (*J* = 10.7 and 6.0 Hz) at δ 4.00 and 4.02, of H-4 appearing as a quintet (*J* = 6.4 Hz) at δ 5.09 and of H_2_-1′ appearing as a multiplet at δ 4.12. Even the EI mass spectrum at 70 eV of **16** shows diagnostic peaks which can be easily associated to 1,1′,4-*O*,*O*′,*O*″-triacetylnigrosphaeritriol (e.g., 274 *m*/*z* [M]^+^, 231 *m*/*z* [M–Ac]^+^, 187 *m*/*z* [M–2Ac]^+^, 128 *m*/*z* [M–2CH_2_–2OAc]^+^, and 43 *m*/*z* [Ac]^+^) (Figure S9).

Compound **2** has a molecular formula C_7_H_14_O_3_, indicating one unsaturation, according to the molecular ion cluster peak at *m*/*z* 147.1016 [M + H]^+^ (calcd for C_7_H_15_O_3_, 147.1021) in the HR-ESI-MS spectrum (Figure S10) and it was identified as an inseparable hemiacetal mixture. The ^1^H NMR spectrum (Figure S11) showed the presence of two doublets at δ 5.58 (*J* = 4.6 Hz) and at δ 5.51 (*J* = 2.8 Hz) in a 62.5:37.5 ratio. These signals were assigned to hemiacetalic groups, which was confirmed by their correlations observed in the HSQC spectrum (Figure S12) to the carbons at δ 99.8 and 101.3 (Figure S13), respectively. COSY spectrum (Figure S14) showed homocorrelations between anomeric proton H-2 of main stereoisomer (**2A**) with H-3 resonating as a multiplet at δ 1.67, from this latter and a multiplet at δ 4.00 of H-6 and from H-6 and Me-7 of a methyl group of a 1-hydroxyethyl side chain.

Moreover, the same spectrum showed a correlation between H-3 and multiplet at δ 2.56 attributed to H-4, from this latter and Me-8 (δ 1.09, *J* = 6.6 Hz) and to triplets attributed to protons of the oxymethylene group at C-5 (δ 4.29, *J* = 8.3 Hz; δ 3.47, *J* = 8.3 Hz) (Figure S14).

The correlations in the HSQC spectrum (Figure S12) allowed the assignment of the chemical shifts to the carbons and related protons. Moreover, the HMBC experiment (Figure S15, Table 2) furnished useful data to solve the structure. The main correlations observed were between C-2 and H-4 and H-5, C-3 and H-4 and H-7, and C-8 and H-3, H-4 and H-5. These data were in accordance with the structure of **2A**.

For isomer **2B**, the same NMR spectra (Figures S11–S15) allowed assigning chemical shifts to all carbons and protons, as reported in Table 2.

The analysis of its nuclear Overhauser effect spectroscopy (NOESY) spectrum showed nuclear Overhauser effects (NOEs) of both the isomers of H-2 with H-3. Furthermore, H-2A proton showed NOE with Me-7 at δ 1.38 and H-2B with Me-7 at δ 1.31 (Figure S16). These observations further supported the structures of the lactols. However, the stereostructure of **2** cannot be determined from these data (see Discussion section for further remarks).

On the basis of spectroscopic data, **2** was characterized as 3-(1-hydroxyethyl)-4-methyltetrahydrofuran-2-ol (Figures S11–S16). The structure of compound **2** was confirmed by preparing its 2,6-*O*,*O*′-diacetyl derivative by reaction with pyridine and acetic anhydride (**17A/17B**, Figure 2). ^1^H and COSY NMR spectra (Figures S17 and S18) showed essentially the diacetyl derivative of stereoisomer **2B** as the main compound (ratio **2A**/**2B** 16.6:83.4). The spectra differed from that of **2B** essentially in the downfield shifts (Δδ 0.73 and 1.21) of doublet and multiplet of H-2 and H-6 observed at δ 6.24 and 5.05, respectively, and in the presence of the singlets of two acetyl groups at δ 2.10 and 2.07. Unfortunately, the chemical shift assignation of **2A** was not possible due the low intensity of the signals. The Total Ion Chromatogram (TIC) of nigrosphaerilactol after trimethylsilylation shows the presence of two well-developed peaks corresponding to comparable EI mass spectra at 70 eV (Figure S19). In fact, the fragmentation patterns of the acquired EI mass spectra extracted at 8.516 min and at 8.969 min could be attributed to epimeric derivatives at C-2 of **2** and their interpretation allows to detect some diagnostic ions (e.g., 275 *m*/*z* [M–Me]^+^, 157 *m*/*z* [M–2Me–CH–OTMS]^+^, 130 *m*/*z* [M–Me–2TMS]^+^, 73 *m*/*z* [TMS]^+^). The TIC of the same fraction after acetylation (Figure S20) shows a single well-developed peak which mass spectrum reports as a fragmentation pattern attributed to 2,6-*O*,*O*′-diacetylnigrosphaerilactol (**17**) showing some diagnostic ions (e.g., 127 *m*/*z* [M–2Me–CH–OAc]^+^, 82 *m*/*z* [M–2Me–2OAc]^+^, and 69 *m*/*z* [M–2Me–CH–2OAc]^+^, 43 *m*/*z* [Ac]^+^).

Nigrosphaerilactone (**3**) was previously described as (3*S*,4*R*,5*R*)-4-hydroxymethyl-3,5-dimethyldihydro-2-furanone [25] (Figure S21). Moreover, nigrosphaerilactone (**3**) was converted into the corresponding 8-*O*-*p*-bromobenzoyl derivative (**18**, Figure 2) by reaction with *p*-bromobenzoyl chloride in CH_3_CN. ^1^H NMR spectrum of **18** differed from that of **3** essentially for the presence of protons of para disubstituted phenyl group resonating at δ 7.89 and 7.64 and for the downfield shift (Δδ 0.66) of two doublets of doublets of H_2_-7 observed at δ 4.50 and 4.42 (Figure S22). The EI mass spectrum at 70 eV (Figure S23) extracted from the well-developed peak at 14.662 min of the TIC, reported in Figure S23A, shows a fragmentation pattern attributable to **18** in which several diagnostic ions can be detected (e.g., 326 *m*/*z* [M]^+^, 200 *m*/*z* [M–2Me–O–Br]^+^, 183 *m/z* [BrPhCO]^+^, 143 *m*/*z* [M–BrPhCO]^+^ and 126 *m*/*z* [M–BrPhCOO]^+^).

The structure and the absolute configuration of compound **3** were confirmed by X-ray diffraction data analysis carried out on the colorless block-shaped crystals obtained by slow evaporation of CHCl_3_/MeOH (5:1) solution at r.t of its 8-*O*-*p*-bromobenzoyl derivative (**18**).

**Figure 3:**
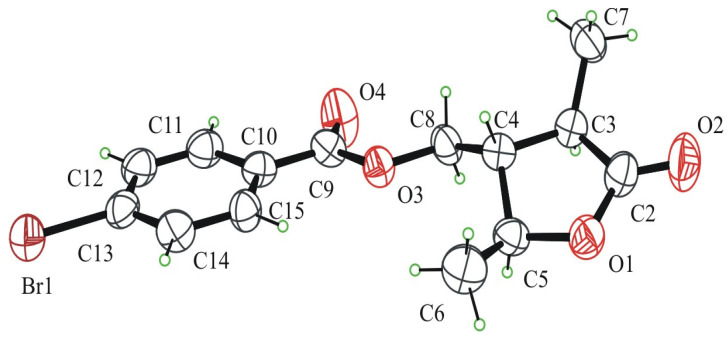
ORTEP view of 8-*O*-*p*-bromobenzoylnigrosphaerilactone (**18**) molecular structure with ellipsoids drawn at 50% probability electron density.

An ORTEP view of compound **18** is shown in Figure 3. Crystal data and refinement details are reported in the Materials and Methods Section and in Table S1 of the Supplementary Materials.

Compound **18** crystallizes in the P2_1_2_1_2_1_ space group with one molecule in the independent unit. All bond lengths and angles are in the normal range, and a selection of geometric parameters are reported in Tables S2 and S3 of Supporting Materials.

In the molecule, the lactone ring adopts the envelope conformation with a C-4 carbon atom at the flap and a mutually trans-disposition of H atoms at the three stereogenic centers C-3, C-4, and C-5 that disclose in the relative 3*R*,4*S*,5*S* configuration. Due to the presence of bromine as a strong anomalous scatterer atom in the molecule, the X-ray crystal structure analysis results allowed us to assign the 3*R*/4*S*/5*S* absolute configuration at the C-3/C-4/C-5 stereogenic centers.

The *p*-Br-benzoate group is all planar with torsion angles at C-8-O-3, O-3-C-9, and C-9-C-10 bonds, very near to 180°. The whole molecule assumes a flat, elongated shape (Table S2 and Figure S24). Normal van der Waals interactions and weak CH^…^O interactions contribute to stabilizing the crystal packing where waved sheets of molecules in the **a** axis direction are formed (Table S1 and Figure S25).

Structures of known compounds (**3**–**8**, Figure 2) were confirmed by comparison of the obtained NMR data with those reported in literature [25,26,27,28,29] (Figures S26–S30). In addition, the GC-MS analysis of chromatographic fractions allowed the identification of several known compounds (**9**–**15**) by comparison of their 70 eV EI mass spectrum with those reported in the commercial library NIST 20 [30] (see Materials and Methods section) (Figures S31–S37).

### 2.3. GC-MS Analysis of Mycelial Crude Extract

**Figure 4:**
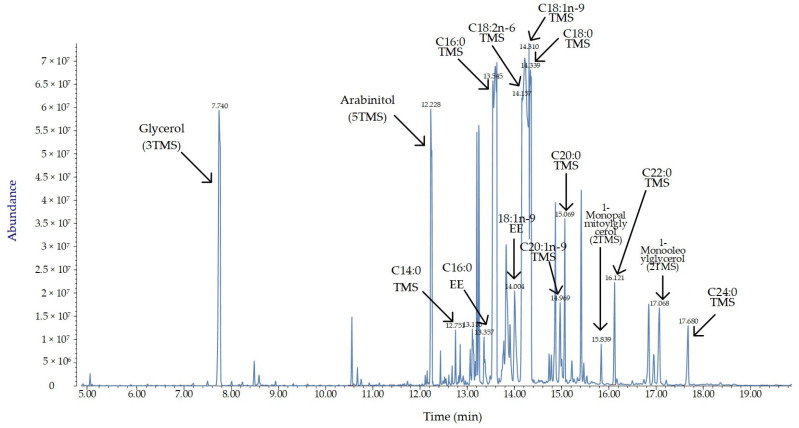
Annotated total ion chromatogram (TIC) acquired after derivatization with BSTFA of the mycelial extract of *Nigrospora sphaerica* associated with buffelgrass (*Cenchrus ciliaris*). EE = ethyl ester; TMS = trimethylsilyl derivative.

The crude extract from mycelium of *N. sphaerica* associated with buffelgrass (*Cenchrus ciliaris*) was analyzed via GC-MS after trimethylsilylation. Figure 4 shows the chromatographic profile of the mycelial extract, and the detected compounds are listed in Table 3 with further details. A number of saturated and unsaturated fatty acids and their esters were identified in the mycelial extract. Moreover, the detection of arabitol turned out to be of special interest due to the several activities associated with this polyol as a product of different fungal species.

## 3. Discussion

*Nigrospora sphaerica* is known for its widespread distribution and can be found in various habitats, including soil, plant debris, and plant surfaces. It is usually reported as a saprophytic fungus, but it is also known to be a plant pathogen, causing diseases in various plants, including fruits and vegetables [31,32]. Some strains of *N. sphaerica* have been studied for the production of novel and diverse bioactive metabolites [32]. The pathogenic association of this fungus with invasive weeds suggests a possible production of phytotoxic compounds, which could help in the development of new weed control strategies [33,34]. In fact, the use of phytopathogenic fungi as biocontrol agents represents a promising path in the search for new weed management alternatives that allow for the reduction of adverse effects of common herbicides [35]. In this work, a screening of secondary metabolites produced in mycelial and culture filtrate extracts of a foliar strain of *N. sphaerica* isolated from buffelgrass (*C. ciliaris*) was conducted after observing an intense activity of the organic extract on buffelgrass seed germination and subsequent seedling growth. Hence, culture filtrate extract was submitted to purification processes, leading to the isolation and identification of several compounds. Subsequent phytotoxicity tests on chromatographic fractions proved that four of them negatively impacted buffelgrass germination to at least some extent. One of the most toxic fractions turned out to be fraction A, which resulted in complete failure of germination, suppressing germination, or aborted germination in all seeds (see Results section). Further investigations of fraction A led to the isolation of two compounds, which could be responsible for the intense toxicity observed, even caused by possible synergistic effects. Most of the metabolites reported in this study were detected in fractions C and L. These fractions still retain a high toxicity but showed a lower toxic effect than fraction A.

Among the metabolites identified in this work, nigrosphaerilactone (**3**) (previously known as (3*S*,4*R*,5*R*)-4-hydroxymethyl-3,5-dimethyldihydro-2-furanone), nigrosporione A (**6**), tyrosol (**8**), adenosine (**10**), uridine (**11**) and ergosta-7,22-dien-3-ol (**16**) have been already reported from *Nigrospora* species, while (*S*)-hydroxybutyrolactone (**4**), lupinlactone (**5**), 2,4-dihydroxy-6-methoxyacetophenone (**7**), 4-hydroxyphenylacetic acid (**9**), ribo-hexonic acid 3-deoxy-γ-lactone (**10**), levoglucosan (**13**), 3-hydroxyisobutyric acid (**14**) were identified for the first time as products of this fungal genus [32]. Along with these known secondary metabolites, the new 3-(hydroxymethyl)-2-methylpentane-1,4-diol, named nigrosphaeritriol (**1**), and the new 3-(1-hydroxyethyl)-4-methyltetrahydrofuran-2-ol, named nigrosphaerilactol (**2**) were structurally elucidated. These compounds exhibit chemical scaffolds that share with known natural products but differ in their substituent patterns. For instance, nigrosphaerilactol is an inseparable hemiacetalic mixture structurally related to the well-known (−)-botryodiplodin, a mycotoxin with a variety of biological activities produced by fungi [36,37,38].

**Figure 5:**
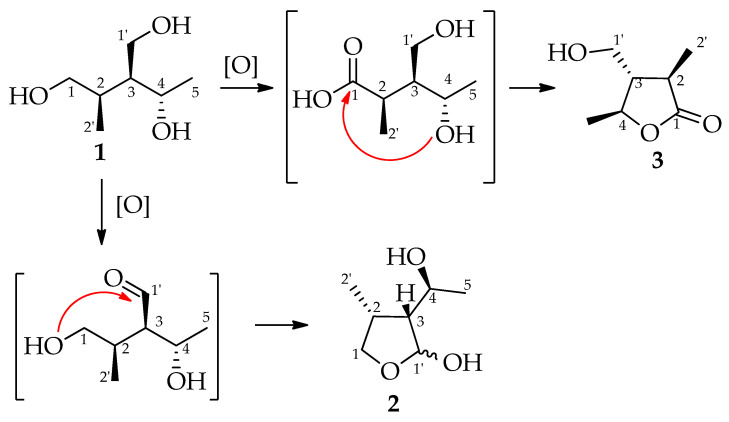
Proposed biosynthetic pathway. Carbon numbers were assigned on the basis of the parental compound (**1**).

Interestingly, as can be seen from Figure 5, the lactol (**2**) and the γ-lactone (**3**) identified in this study seem to be biosynthetically related to nigrosphaeritriol (**1**). In fact, in the proposed mechanism, (3*R*,4*S*,5*S*)-nigrosphaerilactone (**3**) is obtained from **1** by oxidation at C-1 followed by cyclization with the hydroxy group at C-4, while the cyclic hemiacetal nigrosphaerilactol (**2**) is obtained from **1** by oxidation at C-1′ followed by cyclization with the hydroxy group at C-1. Considering this structural relationship, it may be hypothesized the absolute stereochemistry of **1** and **2** on the basis of the absolute stereochemistry of nigrosphaerilactone (**3**) determined in this study by X-ray diffraction analysis, which is (3*R*,4*S*,5*S*)-nigrosphaerilactone. Hence, it can be deduced that **1** can be reported as (2*R*,3*S*,4*S*)-nigrosphaeritriol and **2** can be reported as (2*R*/2*S*, 3*R*,4*R*,6*S*)-nigrosphaerilactol. In addition, the configuration of the hemiacetalic carbon of the two epimers **2A** (2*R*,3*R*,4*R*,6*S*) and **2B** (2*S*,3*R*,4*R*,6*S*) may be deduced by comparing the spectroscopic data of (-)-botryodiplodin, and its analogues and derivatives, with the ones reported for **2** [39].

Among the further metabolites identified in this study, nigrosporione A and lupinlactone were recently characterized. In fact, nigrosporione A (**6**) is a cyclopentanone isolated for the first time from *N. sphaerica* collected from the rice grasshopper displaying antimicrobial activities [27], while lupinlactone (**5**) was recently characterized from culture filtrate extract of the fungus *Colletotrichum lupini* showing phytotoxic effects on different biological systems [30]. In the list of secondary metabolites identified from the strain of *N. sphaerica* under examination, tyrosol (**8**) also appears. It is a phenolic compound commonly produced by plants and microorganisms displaying a number of functions and bioactivities [40,41].

As great lipid-producing microorganisms, fungi from the genus *Nigrospora* are known as oleaginous fungi [42,43,44]. Generally speaking, fatty acids are produced by a variety of fungi because they are important components in terms of structure, membrane constitution, and energy storage [45,46]. The GC-MS analysis conducted on the mycelial extract of *N. sphaerica* showed the presence of saturated and unsaturated fatty acids and their esters. Along with usual fatty acids (e.g., palmitic acid, linoleic acid, stearic acid, and arachidonic acid), several unusual fatty acids (e.g., gondoic acid, behenic acid) were detected. In this respect, gondoic acid (*cis*-11-eicosenoic acid) is a very rare monounsaturated fatty acid reported from plants and only occasionally from fungi [47]. The mycelium of the strain under examination also contains arabitol, a polyol commonly produced by microorganisms, which is an integral part of their normal growth process. The production and yields of polyols are influenced by growth conditions [48,49] because this plays a role in osmo-protection and carbohydrate storage [50].

## 4. Materials and Methods

### 4.1. General Experimental Procedures

Analytical and preparative thin-layer chromatography (TLC) were performed on silica gel (Kieselgel 60, F_254_, 0.25 and 0.5 mm, respectively; Merck, Darmstadt, Germany). Spots were visualized by exposure to UV radiation (254 nm) and by spraying with 10% H_2_SO_4_ in methanol (MeOH) (*v*/*v*), followed by heating at 110 *°*C for 10 min. Sigma-Aldrich Co. (St. Louis, MO, USA) supplied all the solvents. ^1^H and ^13^C NMR spectra were recorded in deuterated chloroform (CDCl_3_) and methanol (CD_3_OD) at 400/100 MHz on Bruker (Karlsruhe, Germany) Anova Advance spectrometer or at 500/125 MHz on Inova 500 (Palo Alto, CA, USA) spectrometers, and the same solvents were used as internal *s*tandard*s*. Optical rotations were measured on a Jasco P-1010 digital polarimeter (Tokyo, Japan). HRESI-TOF mass spectra were measured on an Agilent Technologies ESI-TOF 6230DA instrument in the positive ion mode (Milan, Italy).

### 4.2. Fungal Isolation and Culture Conditions

The *Nigrospora sphaerica* strain used in this study (MR2E2) was obtained from leaf spot lesions on a buffelgrass leaf tissue collection made near La Joya, Hidalgo County, in south Texas, USA, in September 2014. Its characteristic large, black conidial spores permitted provisional morphological identification, which was later confirmed by Sanger sequencing of the ITS region, which is the DNA “barcode” region for routine fungal identification.

Mycelial plugs of stock cultures of the strain of *N. sphaerica* maintained on potato dextrose agar (Oxoid) were used to inoculate 3 L of sterile potato dextrose broth (PDB, Oxoid) in 1 L Erlenmeyer flasks. After 14 days in the stationary phase at laboratory temperature, the cultures were centrifuged and filtrated to separate the liquid phase from mycelium. The resulting culture filtrate and mycelium were lyophilized and stored at −20 °C.

### 4.3. Extraction of Culture Filtrates and Mycelium

The freeze-dried culture filtrate was dissolved in 300 mL of distilled water and then extracted at native pH (≈6) with the same volume of ethyl acetate (EtOAc) three times. The organic extracts were combined, anhydrified with Na_2_SO_4_, and the solvent was evaporated under reduced pressure, yielding a brownish oil residue (371.2 mg).

The freeze-dried mycelium (9.5 g) was homogenized in a mixer with EtOAc (100 mL) three times. The supernatants were combined after centrifugation, dried with anhydrous Na_2_SO_4_, and evaporated under reduced pressure, yielding a yellowish oil residue (835.72 mg).

### 4.4. Isolation of Metabolites from Culture Filtrate Extract

The culture filtrate extract (371.2 mg) was purified by column chromatography (CC) on silica gel eluted (45 cm × 2.5 cm i.d.) with CHCl_3_:*i*-PrOH (9:1, *v*/*v*) and the last fraction was eluted with MeOH. Ten groups of homogenous fractions were collected (A 9.8 mg, B 14.7 mg, C 24.6 mg, D 8.5 mg, E 8.3 mg, F 3.7 mg, G 4.9 mg, H 3.5 mg, I 5 mg, and L 153.4 mg). The residue of the active fractions was further purified (i.e., fractions A, C, and L). Fraction A (9.8 mg) was submitted to a preparative thin layer chromatography (TLC) on silica gel eluted with CHCl_3_:*i*-PrOH (9:1, *v*/*v*) giving nigrosphaerilactol (**2**, 4 mg, *R_f_ 0.37* on TLC on silica gel eluted with CHCl_3_:*i*-PrOH (9:1, *v*/*v*)) and nigrosporione A (**6**, 2 mg, *R_f_* 0.61 on TLC on silica gel eluted with CHCl_3_:*i*-PrOH (9:1, *v*/*v*)). Fraction C (24.6 mg) was submitted to a TLC on silica gel eluted with CHCl_3_:*i*-PrOH (9:1, *v*/*v*), giving nigrosphaerilactone (**3**, 9 mg, *R_f_* 0.58 on TLC on silica gel eluted with CHCl_3_:*i*-PrOH (9:1, *v*/*v*)), (*S*)-hydroxybutyrolactone (**4**, 3.8 mg, *R_f_* 0.63 on TLC on silica gel eluted with CHCl_3_:*i*-PrOH (9:1, *v*/*v*)), lupinlactone (**5**, 3.4 mg, *R_f_* 0.6 on TLC on silica gel eluted with CHCl_3_:*i*-PrOH (9:1, *v*/*v*), tyrosol (**8**, 3 mg, *R_f_* 0.53 on TLC on silica gel eluted with CHCl_3_:*i*-PrOH (9:1, *v*/*v*)).

Fraction L (153.4 mg) was purified via CC on silica gel (40 cm × 1.5 cm i.d.) eluted with EtOAc:MeOH:H_2_O (85:10:5, *v*/*v*/*v*), yielding six homogeneous fractions. The second fraction was purified via preparative TLC, yielding nigrosphaeritriol (**1**, 11.6 mg, *R_f_* 0.33 on TLC on silica gel eluted with EtOAc:MeOH:H_2_O (90:7:3, *v*/*v*/*v*)). The residue of the third fraction (64.0 mg) was further purified by preparative TLC on silica gel, eluted with EtOAc:MeOH:H_2_O (90:7:3, *v*/*v*/*v*), yielding 2,4-dihydroxy-6-methoxyacetophenone (**7**, 6.4 mg, *R_f_* 0.52 on TLC on silica gel eluted with EtOAc:MeOH:H_2_O (90:7:3, *v*/*v*/*v*)), uridine (**11**, 5.5 mg, *R_f_* 0.22 on TLC on silica gel eluted with EtOAc:MeOH:H_2_O (90:7:3, *v*/*v*/*v*)), and adenosine (**10**, 3.5 mg, *R_f_* 0.17 on TLC on silica gel eluted with EtOAc:MeOH:H_2_O (90:7:3, *v*/*v*/*v*)). In addition, GC-MS analysis allowed the identification of 4-hydroxyphenylacetic acid (**9**), 3-hydroxyisobutyric acid (**14**), levoglucosan (**13**), ribo-hexonic acid 3-deoxy-γ-lactone (**12**), and ergosta-7,22-dien-3-ol (**15**) in the fourth TLC fraction (18.0 mg).

*Nigrosphaeritriol* (**1**): 3-(hydroxymethyl)-2-methylpentane-1,4-diol, yellow oil; [α]_D_ +11° (c = 0.5, MeOH); HR-ESI-MS (+): 149.1183 *m*/*z* [M + H]^+^ (calcd for C_7_H_17_O_3_, 149.1178). ^1^H, ^13^C and HMBC NMR data: see Table 1.

*Nigrosphaerilactol* (**2**): 3-(1-hydroxyethyl)-4-methyltetrahydrofuran-2-ol, amorphous solid; HR-ESI-MS (+): 147.1016 *m*/*z* [M + H]^+^ (calcd for C_7_H_15_O_3_, 147.1021). ^1^H, ^13^C, and HMBC NMR data: see Table 2.

### 4.5. Acetylation of Nigrosphaeritriol (***1***)

To nigrosphaeritriol (**1**, 2 mg) dissolved in pyridine (30 μL) was added acetic anhydride (Ac_2_O; 30 μL). The reaction, carried out at room temperature for 12 h, was stopped with MeOH, and the azeotrope formed by the addition of benzene was evaporated at 40 °C in a nitrogen (N_2_) stream. A residue of 1,1′,4-*O*,*O*′,*O*″-triacetylnigrosphaeritriol (**16**) was obtained (4.2 mg, *R_f_* 0.65 on TLC on silica gel eluted with CHCl_3_:*i*-PrOH (95:5, *v*/*v*)).

### 4.6. Acetylation of Nigrosphaerilactol (***2***)

To nigrosphaerilactol (**2**, 1.3 mg) dissolved in pyridine (30 μL) was added acetic anhydride (Ac_2_O; 30 μL). The reaction, carried out at room temperature for 12 h, was stopped with CH_3_OH, and the azeotrope formed by the addition of benzene was evaporated at 40 °C in a nitrogen (N_2_) stream. A residue of 2,6-*O*,*O*′-diacetylnigrospaerilactol (**17**) was obtained (1.9 mg, *R_f_* 0.33 on TLC on silica gel eluted with petroleum ether:acetone (8:2, *v*/*v*)). ^1^H NMR (400 MHz, in CDCl_3_): δ 6.24 (1H, d, *J* = 2.9 Hz, H-2), 5.04 (1H, m, H-6), 4.10 (1H, *J* = 8.0 Hz, H-5a), 3.60 (1H, t, *J* = 8.0 Hz, H-5b), 2.10 (3H, s, CH_3_CO), 2.07 (3H, s,CH_3_CO), 2.03 (1H, m, H-3), 2.00 (1H, m, H-4), 1.28 (3H, d, *J* = 6.5 Hz, C-7), 1.14 (3H, d, *J* = 6.5 Hz, C-8).

### 4.7. Reaction of Nigrosphaerilactone (***3***) with p-Bromobenzoyl Chloride

Nigrosphaerilactone, **3** (8.3 mg), was dissolved in CH_3_CN (1 mL), and 4-dimethylaminopyridine (DMAP) (14.1 mg) and *p*-bromobenzoyl chloride (14.1 mg) were added. The reaction mixture was stirred at room temperature for 4 h and then evaporated under reduced pressure. The residue (34.8 mg) was purified by TLC on silica gel, eluent CHCl_3_/*i*-PrOH (95:5, *v*/*v*), giving derivative 8-*O*-*p*-bromobenzoylnigrosphaerilactone (**18**, 13.4 mg, *R_f_* 0.48 on TLC on silica gel eluted with CHCl_3_:*i*-PrOH (97:3, *v*/*v*)). ^1^H NMR (400 MHz, in CDCl_3_): δ 7.89 (2H, d, *J* = 8.5, Hz, H-2′,6′), 7.64 (2H, d, *J* = 8.5, Hz, H-3′,5′), 4.50 (1H, dd, *J* = 4.5, 11.7 Hz, H-8a), 4.42 (1H, dd, *J* = 5.7, 11.7 Hz, H-8b), 4.39 (1H, m, H-5), 2.61 (1H, dq, *J* = 11.5, 7.1 Hz, H-3), 2.18 (1H, m, H-4), 1.53 (3H, d, *J* = 6.1, Hz, H_3_-6), 1.37 (3H, d, *J =* 7.1 Hz, H_3_-8).

### 4.8. GC-MS Analysis

GC*-*MS data were acquired on the row crude extracts, chromatographic fractions, and pure compounds after trimethylsilylation with *N*,*O*-bis(trimethylsilyl)trifluoroacetamide (BSTFA) (Fluka, Buchs, Switzerland), after acetylation with acetic anhydride in pyridine or after reaction with DMAP and *p*-bromobenzoyl chloride as reported above. GC-MS measurements were performed with an Agilent 6850 GC (Milan, Italy), equipped with an HP-5MS capillary column (5% phenyl methyl poly siloxane stationary phase), coupled to an Agilent 5973 Inert MS detector operated in the full scan mode (*m*/*z* 29–550) at a frequency of 3.9 Hz and with the EI ion source and quadrupole mass filter temperatures kept, respectively, at 200 *°*C and 250 *°*C. Helium was used as carrier gas at a flow rate of 1 mL min*^−^*^1^. The injector temperature was 250 *°*C and the temperature ramp raised the column temperature from 70 *°*C to 280 *°*C: 70 *°*C for 1 min; 10 *°*C min*^−^*^1^ until reaching 170 *°*C; and 30 *°*C min*^−^*^1^ until reaching 280 *°*C. Then, it was held at 280 *°*C for 5 min. The solvent delay was 4 min. Metabolites were identified by comparing their EI mass spectra at 70 eV with spectra of known substances present in the NIST 20 mass spectral library [30]. Moreover, the identification was supported by the Kovats retention index (RI) calculated for each analyte by the Kovats equation, using the standard *n*-alkane mixture in the range C7-C40 (Sigma-Aldrich, Saint Louis, MO, USA).

### 4.9. Crystallographic Structure Determination of 8-O-p-Bromobenzoylnigrosphaerilactone (***18***)

Single crystals of 8-*O*-*p*-bromobenzoylnigrosphaerilactone (**18**) were obtained by slow evaporation of CHCl_3_/MeOH (5:1) of **18**. One selected crystal was mounted at ambient temperature on a Bruker-Nonius KappaCCD diffractometer (graphite monochromated Mo Kα radiation λ = 0.71073 Å, CCD rotation images, thick slices, φ and ω scans to fill the asymmetric unit. A semiempirical absorption correction (multiscan, SADABS [51]) was applied. The structure was solved by direct methods using the SIR97 program [52] and anisotropically refined by the full matrix least-squares method on F^2^ against all independent measured reflections using the SHELXL-2019/2 program [53]. All the hydrogen atoms were introduced in calculated positions and refined according to the riding model with C–H distances in the range 0.93–0.96 Å and with *U*_iso_(H) equal to 1.2*U*_eq_ or 1.5*U*_eq_(C_methyl_) of the carrier atom. Crystals were block-shaped but, being formed by superimposed thin-plated crystals, they were not good quality and weakly diffracting at high θ values. In the presence of bromine strong anomalous scatterer atoms in the compound, the absolute structure parameter is significative (Flack x = 0.044(19) by classical fit to all intensities and x = 0.071(13) by Parsons’ method), and it was possible to assign the absolute configuration at the three stereogenic centers C3/C4/C5 as 3*R*/4*S*/5*S*. The figures were generated using ORTEP-3 [54] and Mercury 4.0 [55]. Details on the crystal structure are reported in the Supplementary Materials.

*Crystallographic Data:* empirical formula C_14_ H_15_ Br O_4_; formula weight 327.17 g·mol^−1^; orthorhombic, P 2_1_2_1_2_1_, *a* = 7.3450(6), *b* = 11.486(2), *c* = 16.5930(19)Å; V = 1399.9(3) Å^3^; Z = 4, D_x_ = 1.552 Mg/m^3^, Absolute structure parameter = 0.071(13).

All crystallographic data for 8-*O*-*p*-bromobenzoylnigrosphaerilactone (**18**) were deposited in the Cambridge Crystallographic Data Centre with deposition number CCDC 2322188. These data can be obtained free of charge from www.ccdc.cam.ac.uk/data_request/cif.

### 4.10. Seed Germination and Seedling Growth Bioassay

Seeds of buffelgrass (*C. ciliaris*) were used for this assay, which was carried out with the culture filtrate extract and subsequently with chromatographic fractions A, B, C, and L. These were first dissolved in DMSO and then brought to a concentration of 2 mg mL^−1^ with distilled water (final DMSO concentration 2%). 250 μL of each solution was pipetted into each of three 3.5 cm Petri dishes onto the surface of one filter paper. Seeds were incubated in 2% DMSO in the control treatment. Six buffelgrass seeds were arranged onto the surface of each filter paper in a pattern that made it possible to track individual seeds. Petri dishes were sealed with parafilm to retard moisture loss and incubated at 25 °C with a 12:12 h photoperiod. Germination, defined as visible radicle emergence, was scored each day for 7 days, and germination day was tracked individually for each seed. Three days after germination, the coleoptile and radicle length for each seedling was recorded using electronic calipers. As very few seeds other than control seeds germinated within 7 days, data on seedling radicle and coleoptile elongation are not reported here. The remaining ungerminated seeds were left in incubation for an additional 7 days to quantify delayed germination. Seeds were scored as germinated within 7 days, germinated after delay, ungerminated, or with aborted germination (coleorhiza exserted but with no growth of the radicle or coleoptile). Ungerminated seeds and seeds with aborted germination were pooled for analysis as seeds with failed germination.

### 4.11. Statistical Analysis

The data were analyzed using SAS 9.4 (SAS Institute, Cary, NC, USA) Proc ANOVA for a completely randomized design with three replicates per treatment to obtain means separations evaluating (1) the effects of raw culture filtrate and (2) the effects of the four chromatographic fractions on each of the three response variables. The two experiments were analyzed separately. The response variables in each analysis were the fraction of seeds germinating within 7 days, the fraction with delayed germination (>7 days), and the fraction that failed to germinate normally. The Ryan–Einot–Gabriel–Welsch multiple range test at *p* < 0.05) procedure at *p* < 0.05 was used for means separations.

## 5. Conclusions

The fungus *Nigrospora sphaerica* represents a promising agent for buffelgrass biocontrol that allows for the reduction of adverse effects of synthetic herbicides. This article describes the isolation and structural elucidation of a new triol, named nigrosphaeritriol (**1**), and a new lactol, named nigrosphaerilactol (**2**), along with several known compounds from the culture filtrate extract of a foliar strain of *N. sphaerica* isolated from buffelgrass which showed a strong phytotoxic activity on buffelgrass seed germination and subsequent seedling growth. In this work, the absolute stereochemistry of (3*R*,4*S*,5*S*)-nigrosphaerilactone (**3**) was determined for the first time by X-ray diffraction analysis. Moreover, considering the structural relationship among **1**–**3**, the absolute stereochemistry of **1** and **2** was inferred from that of **3**. In addition, a number of saturated and unsaturated fatty acids and their esters, which are important components of the biomass of the microbial communities, were identified in the mycelial extract via GC-MS.

## Figures and Tables

**Table 1:** ^1^H, ^13^C, and HMBC NMR data of nigrosphaeritriol (**1**) *^a^*^,*b*^ and ^1^H NMR data of its triacetyl derivative (**16**) *^a^*.

	1			16
Position	δ_C_ *^c^*	δ_H_ (*J* in Hz)	HMBC *^c^*	δ_H_ (*J* in Hz)
1	66.2	3.55 (1H) dd (10.7, 6.1) 3.51 (1H) dd (10.7, 5.9)	H-3, H_3_C-2′	4.03 (1H) dd (10.7, 6.0) 4.14 (1H) dd (10.7, 6.0)
2	34.0	2.05 (1H) m	H_2_C-1, H-3, H-4, H_2_C-1′, H_3_C-2′	2.17 (1H) m
3	48.7	1.60 (1H) m	H_2_C-1, H-4, H_3_C-5, H_2_C-1′, H_3_C-2′	1.95 (1H) m
4	67.1	3.90 (1H) quint (6.4)	H_3_C-5, H_2_C-1′	5.13 (1H) quint (6.4)
5	20.1	1.24 (3H) d (6.4)	H-3, H-4	1.27 (3H) d (6.4)
1′	59.7	3.62 (2H) m	H-3, H-4	4.12 (2H) m
2′	12.3	0.98 (3H) d (7.1)	H_2_C-1, H-3	0.99 (3H) d (7.1)
C-OAc *^d^*				2.10 (3H) s
C-OAc *^d^*				2.08 (3H) s
C-OAc *^d^*				2.07 (3H) s

*^a^*The chemical shifts are in δ values (ppm) from TMS. *^b^*2D ^1^H, ^1^H (COSY) ^13^C, and ^1^H (HSQC) NMR experiments delineated the correlations of all the protons and the corresponding carbons. *^c^*HMBC correlations are from the stated carbon to the indicated proton(s). *^d^* The attributions of these signals could be inverted.

**Table 2:** ^1^H, ^13^C and HMBC NMR data of nigrosphaerilactol (**2**) *^a^*^,*b*^.

		2A		2B		
Position	δ_C_ *^c^*	δ_H_ (*J* in Hz)	HMBC *^c^*	δ_C_ *^c^*	δ_H_ (*J* in Hz)	HMBC *^c^*
2	99.8	5.58 (1H) d (4.6)	H-4, H-5	101.3	5.51 (1H) d (2.8)	H-5
3	57.2	1.67 (1H) m	H-4, H-7	62.1	1.66 (1H) m	H-4, H-7
4	32.1	2.54 (1H) m	H-3, H-6	36.0	2.08 (1H) m	H-3, H-6
5	74.6	4.29 (1H) t (8.3) 3.47 (1H) t (8.3)	H-2, H-3, H-8	73.7	4.04 (1H) t (9.2) 3.68 (1H) t (9.2)	H-2, H-3
6	65.7	4.00 (1H) m	H-2	68.0	3.83 (1H) m	H-2
7	22.6	1.38 (3H) d (6.5)	H-3, H-6	22.6	1.31 (3H) d (6.3)	H-3, H-6
8	16.8	1.09 (3H) d (6.6)	H-3, H-4, H-5	16.8	1.13 (3H) d (6.4)	H-3, H-4, H-5

*^a^* The chemical shifts are in δ values (ppm) from TMS. *^b^* 2D ^1^H, ^1^H (COSY) ^13^C, ^1^H (HSQC) NMR experiments delineated the correlations of all the protons and the corresponding carbons. *^c^* HMBC correlations are from the stated carbon to the indicated proton(s).

**Table 3:** GC-MS profile of the crude extract after trimethylsilylation of the mycelium of *Nigrospora sphaerica* associated to *Cenchrus ciliaris*. EE = ethyl ester; TMS = trimethylsilyl derivative.

	Name	Retention Time (Min)	Retention Index	Area %
	Glycerol (3TMS)	7.74	1287	23.732
	Arabitol (5 TMS)	12.228	1746	17.01
C14:0 TMS	Myristic acid (TMS)	12.751	1850	1.243
C16:0 EE	Palmitic acid (EE)	13.357	1994	2.302
C16:0 TMS	Palmitic acid (TMS)	13.545	2045	5.272
18:1n-9 EE	Oleic acid (EE)	14.004	2173	5.651
C18:2n-6 TMS	Linoleic acid (TMS)	14.157	2210	5.058
C18:1n-9 TMS	Oleic acid (TMS)	14.31	2225	7.519
C18:0 TMS	Stearic acid (TMS)	14.339	2240	11.689
C20:1n-9 TMS	Gondoic acid (TMS)	14.969	2420	2.678
C20:0 TMS	Arachidic acid (TMS)	15.069	2442	4.294
	1-Monopalmitoylglycerol (2TMS)	15.839	2592	1.228
C22:0 TMS	Behenic acid (TMS)	16.121	2637	4.089
	1-Monooleoylglycerol (2TMS)	17.068	2769	4.904
C24:0 TMS	Lignoceric acid (TMS)	17.68	2838	3.331

## Data Availability

The data that support the findings of this study are available from the corresponding author upon reasonable request.

## Supplementary Materials

The following supporting information can be downloaded at: https://www.mdpi.com/article/10.3390/molecules29020438/s1. Figure S1. HR-ESI-MS spectrum of nigrosphaeritriol (**1**) recorded in positive modality. Figure S2. ^13^C NMR spectrum of nigrosphaeritriol (**1**) recorded at 100 MHz in CD_3_OD. Figure S3. ^1^H NMR spectrum of nigrosphaeritriol (**1**) recorded at 400 MHz in CD_3_OD. Figure S4. COSY spectrum of nigrosphaeritriol (**1**) recorded at 400 MHz in CD_3_OD. Figure S5. HSQC spectrum of nigrosphaeritriol (**1**) recorded at 400 MHz in CD_3_OD. Figure S6. HMBC spectrum of nigrosphaeritriol (**1**) recorded at 400 MHz in CD_3_OD. Figure S7. GC-MS analysis of purified chromatographic fraction containing nigrosphaeritriol (**1**) after trimethylsilylation with BSTFA: (A) Total ion chromatogram and (B) EI mass spectrum at 70 eV extracted at 10.557 min (RI = 1515). Figure S8. ^1^H NMR spectrum of 1,1′,4-*O*,*O*′,*O*″-triacetylnigrosphaerilactol (**17**), recorded at 400 MHz in CDCl_3_. Figure S9. GC-MS analysis of purified chromatographic fraction containing nigrosphaeritriol (1) after acetylation (1,1′,4-*O*,*O*′,*O*″-triacetylnigrosphaerilactol (**16**)): (A) Total ion chromatogram and (B) EI mass spectrum at 70 eV extracted at 11.786 min (RI = 1671). Figure S10. HR-ESI-MS spectrum of nigrosphaerilactol (**2**) recorded in positive modality. Figure S11. ^1^H NMR spectrum of nigrosphaerilactol (**2**) recorded at 500 MHz in CDCl_3_. Figure S12. HSQC spectrum of nigrosphaerilactol (**2**) recorded at 500 MHz in CDCl_3_. Figure S13. ^13^C NMR spectrum of nigrosphaerilactol (**2**) recorded at 100 MHz in CDCl_3_. Figure S14. COSY spectrum of nigrosphaerilactol (**2**) recorded at 500 MHz in CDCl_3_. Figure S15. HMBC spectrum of nigrosphaerilactol (**2**) recorded at 500 MHz in CDCl_3_. Figure S16. NOESY spectrum of nigrosphaerilactol (**2**) recorded at 500 MHz in CDCl_3_. Figure S17. ^1^H NMR spectrum of 2,6-*O*,*O*’-diacetylnigrosphaerilactol (**17**) recorded at 400 MHz in CDCl_3_. Figure S18. COSY spectrum of 2,6-*O*,*O*’-diacetylnigrosphaerilactol (**17**) recorded at 400 MHz in CDCl_3_. Figure S19. GC-MS analysis of purified chromatographic fraction containing nigrosphaerilactol (**2**) after trimethylsilylation with BSTFA: (A) total ion chromatogram; (B) EI mass spectrum at 70 eV extracted at 8.516 min (RI =1346); (C) EI mass spectrum at 70 eV extracted at 8.969 min (RI = 1381). Figure S20. GC-MS analysis of purified chromatographic fraction containing nigrosphaerilactol (**2**) after acetylation (2,6-*O*,*O*′-diacetylnigrospaerilactol, **17**): (A) total ion chromatogram and (B) EI mass spectrum at 70 eV extracted at 9.939 min (RI = 1459). Figure S21. ^1^H NMR spectrum of nigrosphaerilactone (**3**) recorded at 400 MHz in CDCl_3_. Figure S22. ^1^H NMR spectrum of *8*-*O*-*p*-bromobenzoylnigrosphaerilactone (**18**) recorded at 400 MHz in CDCl_3_. Figure S23. GC-MS analysis of purified chromatographic fraction containing nigrosphaerilactone (**2**) with *p*-bromobenzoyl chloride giving *8*-*O*-*p*-bromobenzoynigrosphaerilactone (**18**): (A) total ion chromatogram and (B) EI mass spectrum at 70 eV extracted at 14.662 min (RI = 2348). Table S1. Crystal data and structure refinement for 8-*O*-*p*-bromobenzoylnigrosphaerilactone (**18**). Table S2. Selected bond lengths and angles with standard deviations for 8-*O*-*p*-bromobenzoylnigrosphaerilactone (**18**). Table S3. Hydrogen bonds for 8-*O*-*p*-bromobenzoylnigrosphaerilactone (**18**). Figure S24. Perspective view of the molecule in the edge of phenyl plane in the Capped Sticks style (up) and Spacefill style (down) showing the flat elongated shape of the molecule. Figure S25. Crystal packing viewed along the b-axis shows waved sheets of molecules. Contacts shorter than the sum of vdW radii are drawn as cyan and red dashed lines. Figure S26. ^1^H NMR spectrum of (*S*)-hydroxybutyrolactone (**4**) recorded at 400 MHz in CDCl_3_. Figure S27. ^1^H NMR spectrum of lupinlactone (**5**) recorded at 400 MHz in CDCl_3_. Figure S28. ^1^H NMR spectrum of nigrosporione A (**6**) recorded at 400 MHz in CDCl_3_. Figure S29. ^1^H NMR spectrum of 2,4-dihydroxy-6-methoxyacetophenone (**7**) recorded at 400 MHz in CD_3_OD. Figure S30. ^1^H NMR spectrum of tyrosol (**8**) recorded at 400 MHz in CD_3_OD. Figure S31. EI mass spectrum at 70 eV of 4-hydroxyphenylacetic acid (**9**), 2TMS (RI = 1581). TMS = trimethylsilyl group. Figure S32. EI mass spectrum at 70 eV of adenosine (**10**), 4TMS (RI = 2670). Figure S33. EI mass spectrum at 70 eV of uridine (**11**), 3TMS (RI = 2496). Figure S34. EI mass spectrum at 70 eV of ribo-hexonic acid 3-deoxy-γ-lactone (**12**), 3TMS (RI = 1795). Figure S35. EI mass spectrum at 70 eV of levoglucosan (**13**), 3TMS (RI = 1726). TMS = trimethylsilyl group. Figure S36. EI mass spectrum at 70 eV of 3-hydroxyisobutyric acid (**14**), 2TMS (RI = 1167). Figure S37. EI mass spectrum at 70 eV of ergosta-7,22-dien-3-ol (**15**), TMS (RI = 2589).
