# Supplementary material for: Screening of Secondary Metabolites Produced by Nigrospora sphaerica Associated with the Invasive Weed Cenchrus ciliaris Reveals Two New Structurally Related Compounds
Source: Molecules. 2024 Jan 16;29(2):438. doi: 10.3390/molecules29020438 (PMC10818434; doi:10.3390/molecules29020438)
Supplement: Supplementary file 1 [file molecules-29-00438-s001.zip › molecules-2822894-supplementary.pdf]

Supplementary Materials

Screening of Secondary Metabolites Produced by *Nigrospora sphaerica* Associated with the Invasive Weed *Cenchrus ciliaris* Reveals Two New Structurally Related Compounds

Maria Michela Salvatore [1,†], Maria Teresa Russo [1,†], Susan Meyer [2], Angela Tuzi [1], Marina DellaGreca [1], Marco Masi [1, 3,*] and Anna Andolfi [1,3]

[1] Department of Chemical Sciences, University of Naples Federico II, 80126 Naples, Italy; mariamichela.salvatore@unina.it (M.M.S.), mariateresa.russo2@unina.it (M.T.R), angela.tuzi@unina.it (A.T.), dellagre@unina.it (M.DG.); andolfi@unina.it (A.A.);

[2] Department of Geosciences, Southern Utah University, Cedar City, UT 84721, USA; susanmeyer@suu.edu (S.M.)

[3] BAT Center-Interuniversity Center for Studies on Bioinspired Agro-Environmental Technology, University of Naples Federico II, 80055 Portici (NA), Italy

* Correspondence: marco.masi@unina.it (M.M.)

† These authors contributed equally to this work.

Abstract: In the search for new alternative biocontrol strategies, phytopathogenic fungi could represent a new frontier for weed management. In this respect, as part of our ongoing work aiming at using fungal pathogens as alternative to common herbicides, the foliar pathogen *Nigrospora sphaerica* has been evaluated to control buffelgrass (*Cenchrus ciliaris*). In particular, in this work the isolation and structural elucidation of two new biosynthetically related metabolites, named nigrosphaeritriol (3-(hydroxymethyl)-2-methylpentane-1,4-diol) and nigrosphaerilactol (3-(1-hydroxyethyl)-4-methyltetrahydrofuran-2-ol), from the phytotoxic culture filtrate extract were described, along with the identification of several known metabolites. Moreover, the absolute stereochemistry of (3*R*,4*S*,5*S*)-nigrosphaerilactone, previously reported as (3*S*,4*R*,5*R*)-4-hydroxymethyl-3,5-dimethyldihydro-2-furanone, was determined for the first time by X-ray diffraction analysis. Considering their structural relationship, the determination of the absolute stereochemistry of nigrosphaerilactone allowed us to hypothesize the absolute stereochemistry of nigrosphaeritriol and nigrosphaerilactol.

Keywords: Specialized Metabolites; Buffelgrass; Phytopathogen; Bioherbicide; Metabolomics.

Contents

Figure S1. HR-ESI-MS spectrum of nigrosphaeritriol (**1**) recorded in positive modality.

Figure S2. ^{13}C NMR spectrum of nigrosphaeritriol (**1**) recorded at 100 MHz in CD_3OD.

Figure S3. ^{1}H NMR spectrum of nigrosphaeritriol (**1**) recorded at 400 MHz in CD_3OD.

Figure S4. COSY spectrum of nigrosphaeritriol (**1**) recorded at 400 MHz in CD_3OD.

Figure S5. HSQC spectrum of nigrosphaeritriol (**1**) recorded at 400 MHz in CD_3OD.

Figure S6. HMBC spectrum of nigrosphaeritriol (**1**) recorded at 400 MHz in CD_3OD.

Figure S7. GC-MS analysis of purified chromatographic fraction containing nigrosphaeritriol (**1**) after trimethylsilylation with BSTFA: A) Total ion chromatogram and B) EI mass spectrum at 70 eV extracted at 10.557 min (RI = 1515). TMS = trimethylsilyl group.

Figure S8. ^{1}H NMR spectrum of 1,1′,4-*O*,*O*′,*O*′′-triacetylnigrosphaerilactol (**17**), recorded at 400 MHz in $CDCl_3$.

Figure S9. GC-MS analysis of purified chromatographic fraction containing nigrosphaeritriol (1) after acetylation (1,1′,4-*O*,*O*′,*O*′′-triacetylnigrosphaerilactol (**16**)): A) Total ion chromatogram and B) EI mass spectrum at 70 eV extracted at 11.786 min (RI = 1671). Ac = acetyl group.

Figure S10. HR-ESI-MS spectrum of nigrosphaerilactol (**2**) recorded in positive modality.

Figure S11. ^{1}H NMR spectrum of nigrosphaerilactol (**2**) recorded at 500 MHz in $CDCl_3$.

Figure S12. HSQC spectrum of nigrosphaerilactol (**2**) recorded at 500 MHz in $CDCl_3$.

Figure S13. ^{13}C NMR spectrum of nigrosphaerilactol (**2**) recorded at 100 MHz in $CDCl_3$.

Figure S14. COSY spectrum of nigrosphaerilactol (**2**) recorded at 500 MHz in $CDCl_3$.

Figure S15. HMBC spectrum of nigrosphaerilactol (**2**) recorded at 500 MHz in $CDCl_3$.

Figure S16. NOESY spectrum of nigrosphaerilactol (**2**) recorded at 500 MHz in $CDCl_3$.

Figure S17. ^{1}H NMR spectrum of 2,6-*O*,*O*′-diacetylnigrosphaerilactol (**17**) recorded at 400 MHz in $CDCl_3$.

Figure S18. COSY spectrum of 2,6-*O*,*O*′-diacetylnigrosphaerilactol (**17**) recorded at 400 MHz in $CDCl_3$.

Figure S19. GC-MS analysis of purified chromatographic fraction containing nigrosphaerilactol (**2**) after trimethylsilylation with BSTFA: A) Total ion chromatogram; B) EI mass spectrum at 70 eV extracted at 8.516 min (RI =1346); C) EI mass spectrum at 70 eV extracted at 8.969 min (RI = 1381). TMS = trimethylsilyl group.

Figure S20. GC-MS analysis of purified chromatographic fraction containing nigrosphaerilactol (**2**) after acetylation (2,6-*O*,*O*′-diacetylnigrospaerilactol, **17**): A) Total ion chromatogram and B) EI mass spectrum at 70 eV extracted at 9.939 min (RI = 1459). Ac = acetyl group.

Figure S21. ^{1}H NMR spectrum of nigrosphaerilactone (**3**) recorded at 400 MHz in $CDCl_3$.

Figure S22. ^{1}H NMR spectrum of *8-O-p*-bromobenzoylnigrosphaerilactone (**18**) recorded at 400 MHz in $CDCl_3$.

Figure S23. GC-MS analysis of purified chromatographic fraction containing nigrosphaerilactone (**2**) with *p*-bromobenzoyl chloride giving *8-O-p*-bromobenzoynigrosphaerilactone (**18**): A) Total ion chromatogram and B) EI mass spectrum at 70 eV extracted at 14.662 min (RI = 2348).

Table S1. Crystal data and structure refinement for 8-*O*-*p*-bromobenzoylnigrosphaerilactone (**18**).

Table S2. Selected bond lengths and angles with standard deviations for 8-*O*-*p*-bromobenzoylnigrosphaerilactone (**18**).

Table S3. Hydrogen bonds for 8-*O*-*p*-bromobenzoylnigrosphaerilactone (**18**).

Figure S24. Perspective view of the molecule in the edge of phenyl plane in the Capped Sticks style (up) and Spacefill style (down) showing flat elongated shape of the molecule.

Figure S25. Crystal packing viewed along **b** axis showing waved sheets of molecules. Contacts shorter than the sum of vdW radii are drawn as cyan and red dashed lines.

Figure S26. ^{1}H NMR spectrum of (*S*)-hydroxybutyrolactone (**4**) recorded at 400 MHz in $CDCl_3$.

Figure S27. ^{1}H NMR spectrum of lupinlactone (**5**) recorded at 400 MHz in $CDCl_3$.

Figure S28. ^{1}H NMR spectrum of nigrosporione A (**6**) recorded at 400 MHz in $CDCl_3$.

Figure S29. ^{1}H NMR spectrum of 2,4-dihydroxy-6-methoxyacetophenone (**7**) recorded at 400 MHz in CD_3OD.

Figure S30. ^{1}H NMR spectrum of tyrosol (**8**) recorded at 400 MHz in CD_3OD.

Figure S31. EI mass spectrum at 70 eV of 4-hydroxyphenylacetic acid (**9**), 2TMS (RI = 1581). TMS = trimethylsilyl group.

Figure S32. EI mass spectrum at 70 eV of adenosine (**10**), 4TMS (RI = 2670). TMS = trimethylsilyl group.

Figure S33. EI mass spectrum at 70 eV of uridine (**11**), 3TMS (RI = 2496). TMS = trimethylsilyl group.

Figure S34. EI mass spectrum at 70 eV of ribo-hexonic acid 3-deoxy-γ-lactone (**12**), 3TMS (RI = 1795). TMS = trimethylsilyl group.

Figure S35. EI mass spectrum at 70 eV of levoglucosan (**13**), 3TMS (RI = 1726). TMS = trimethylsilyl group.

Figure S36. EI mass spectrum at 70 eV of 3-hydroxyisobutyric acid (**14**), 2TMS (RI = 1167). TMS = trimethylsilyl group.

Figure S37. EI mass spectrum at 70 eV of ergosta-7,22-dien-3-ol (**15**), TMS (RI = 2589). TMS = trimethylsilyl group.

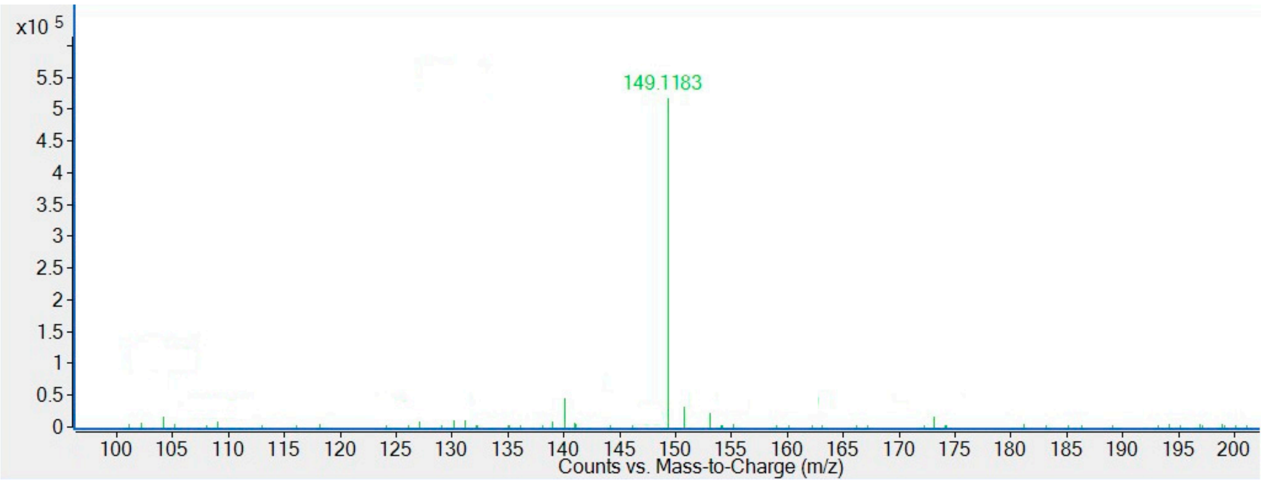

Figure S1. HR-ESI-MS spectrum of nigrosphaeritriol (**1**) recorded in positive modality.

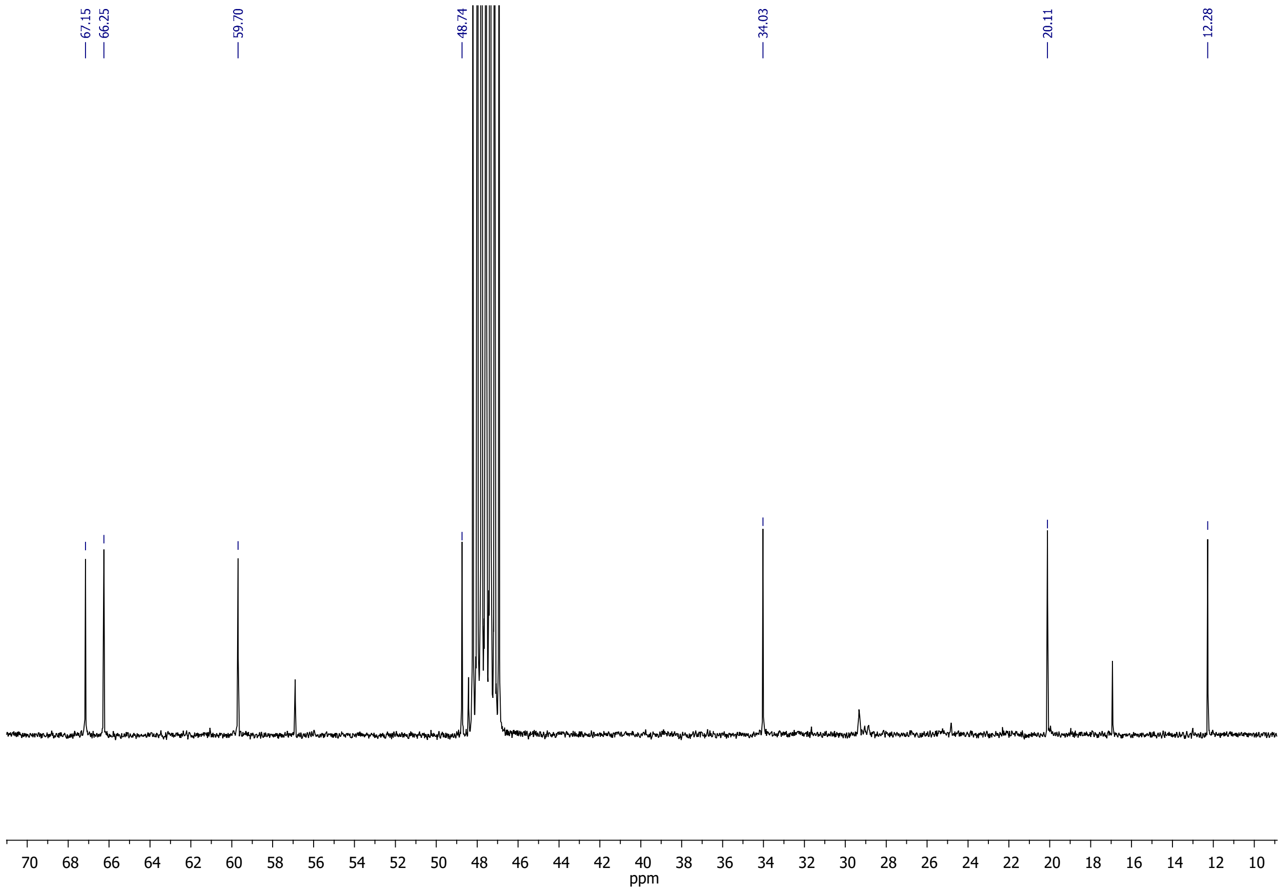

Figure S2. ^{13}C NMR spectrum of nigrosphaeritriol (**1**) recorded at 100 MHz in CD_3OD.

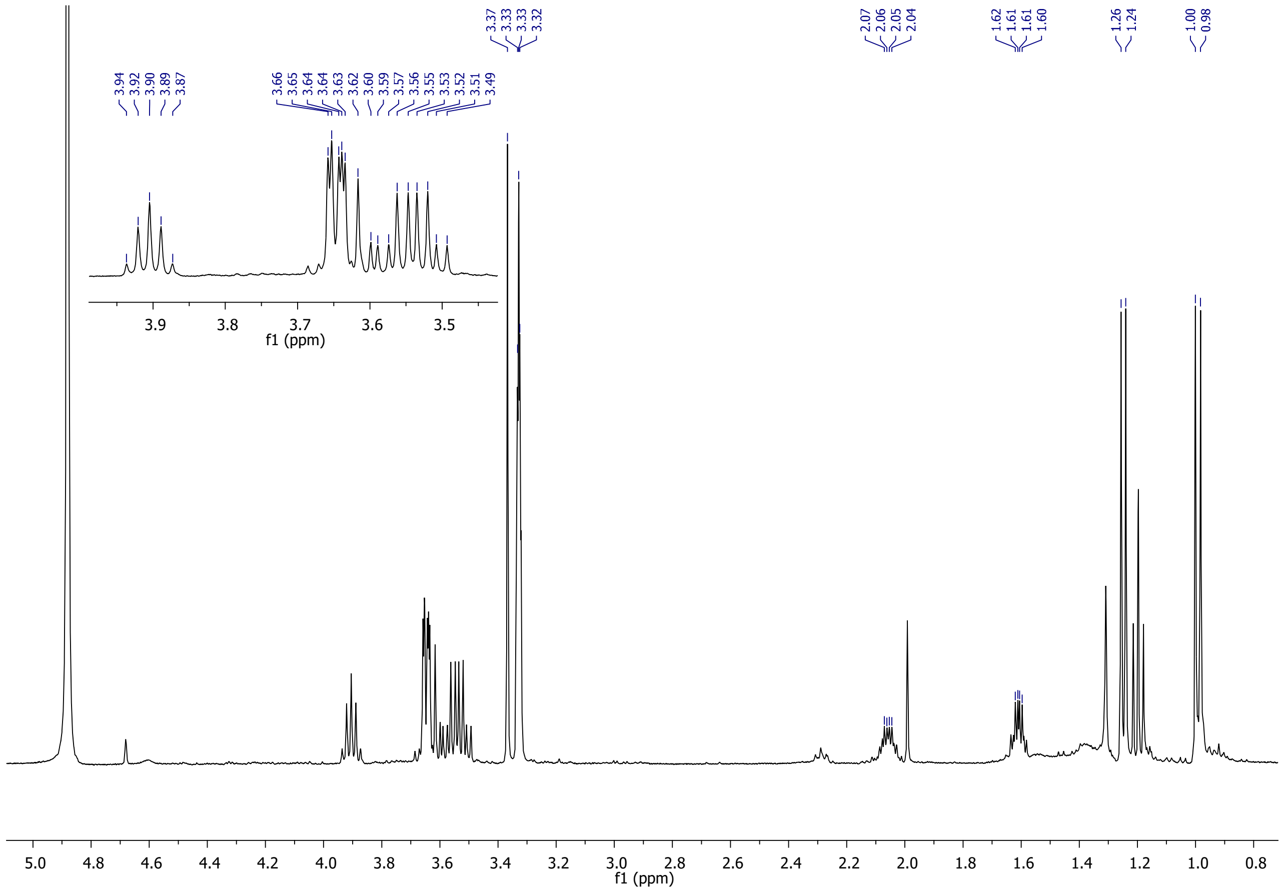

Figure S3. ^{1}H NMR spectrum of nigrosphaeritriol (**1**) recorded at 400 MHz in CD_3OD.

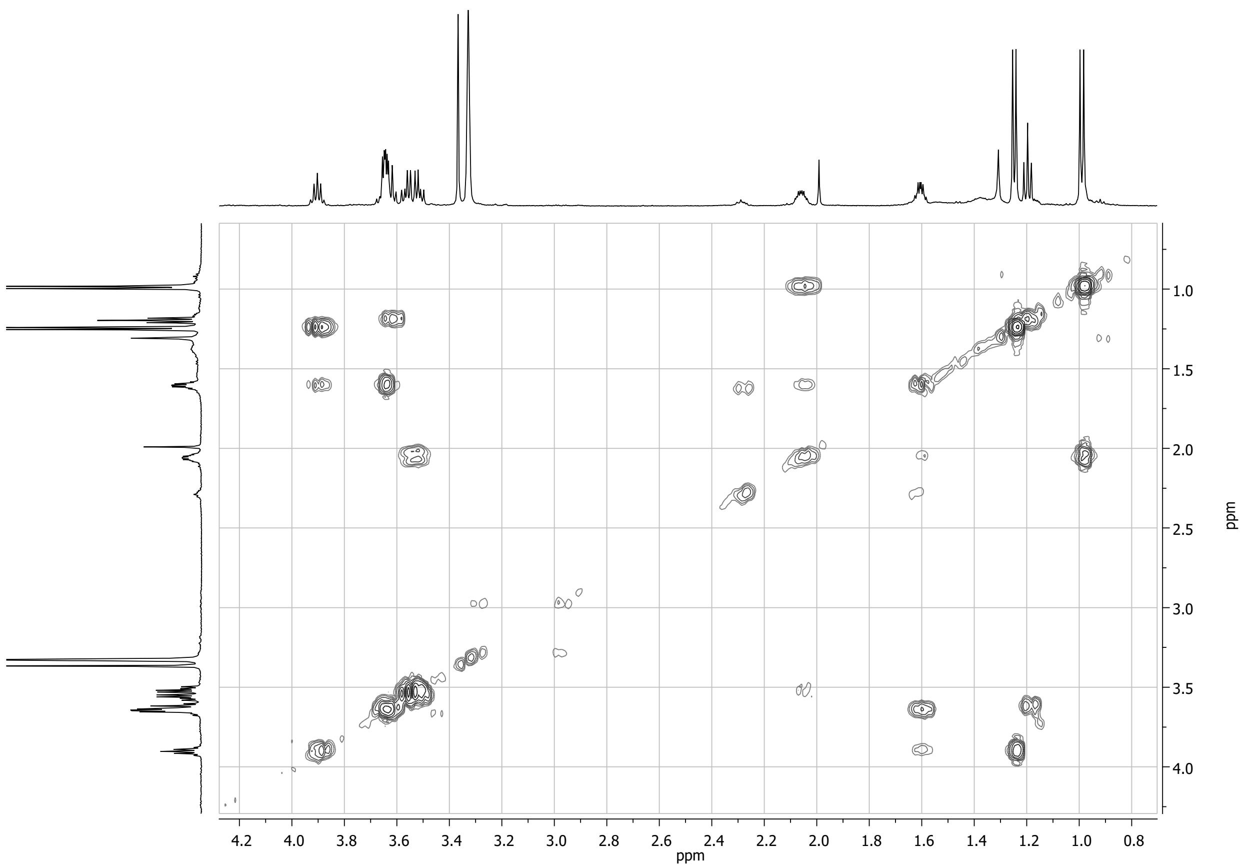

Figure S4. COSY spectrum of nigrosphaeritriol (**1**) recorded at 400 MHz in CD_3OD.

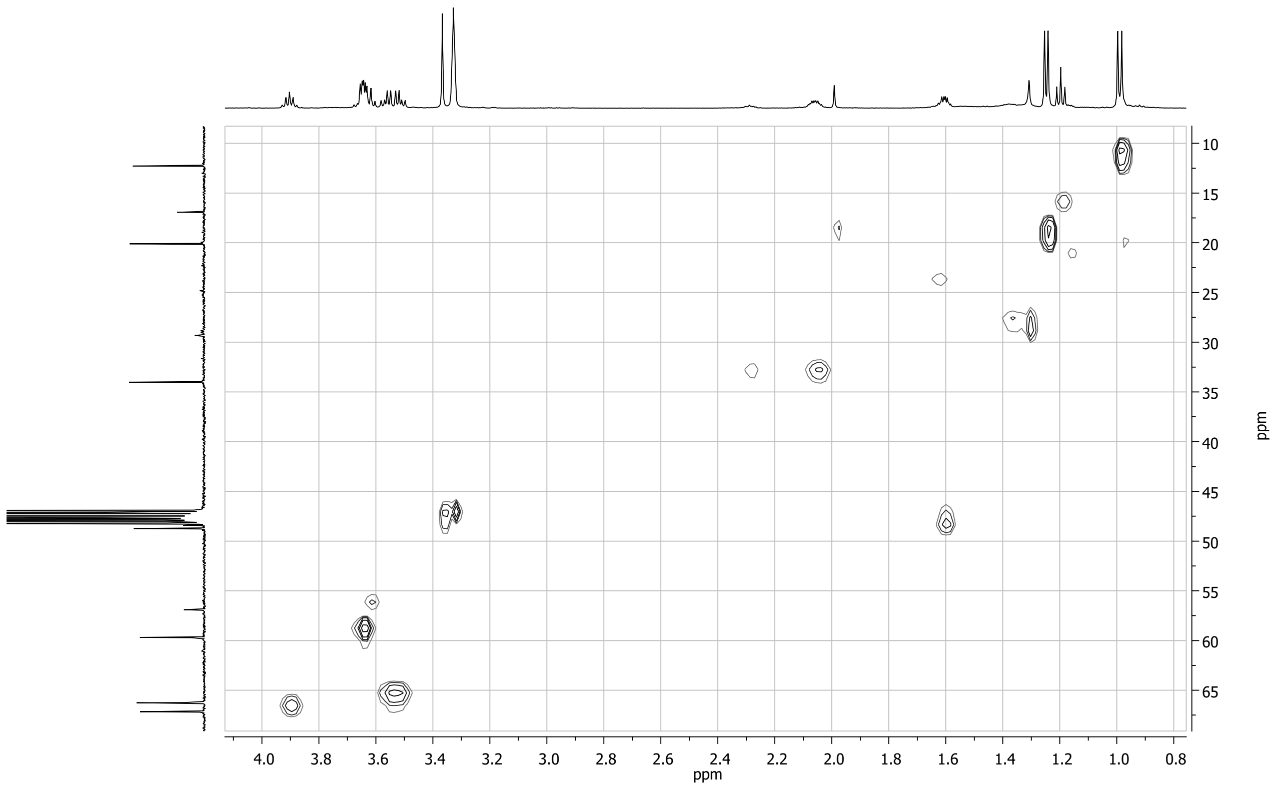

Figure S5. HSQC spectrum of nigrosphaeritriol (**1**) recorded at 400 MHz in CD_3OD.

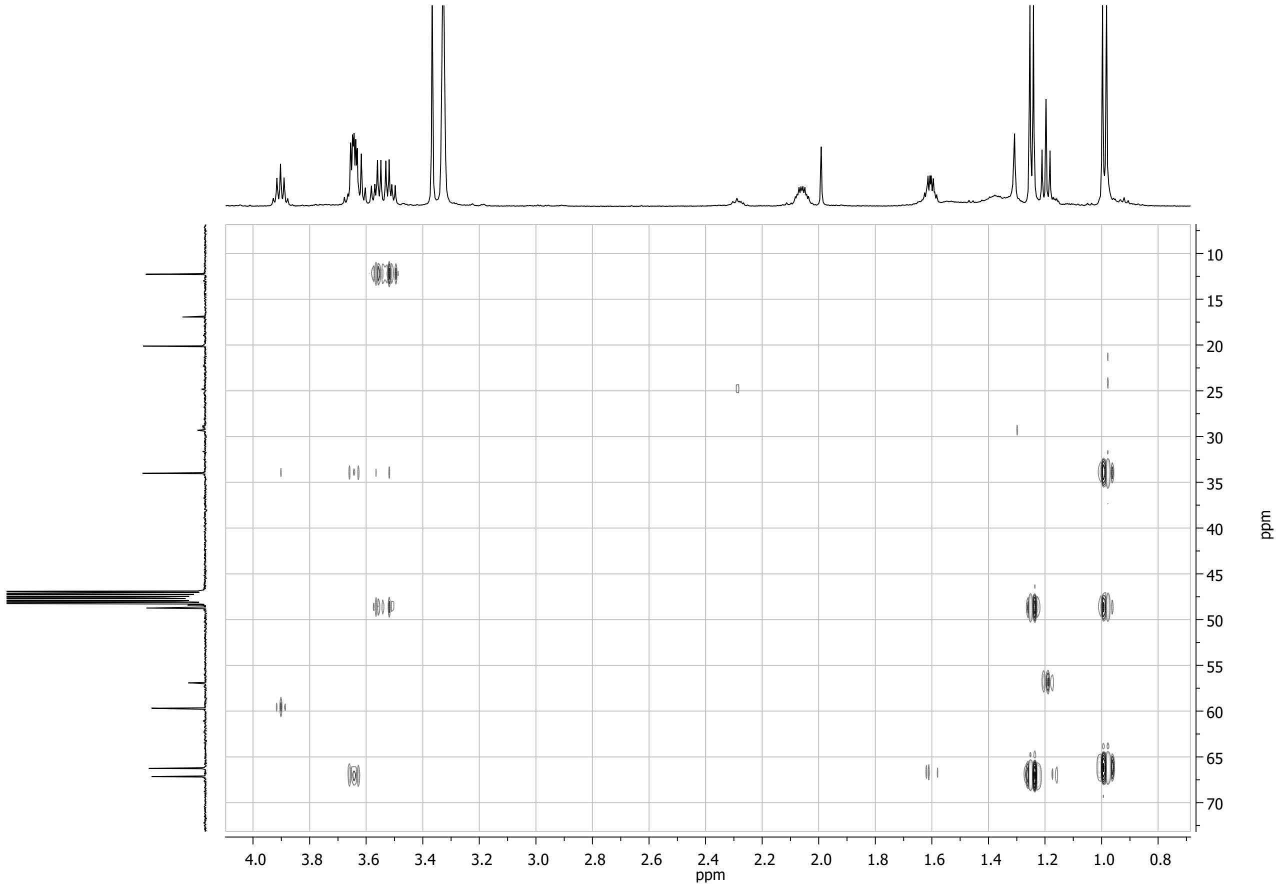

Figure S6. HMBC spectrum of nigrosphaeritriol (**1**) recorded at 400 MHz in CD_3OD.

A)

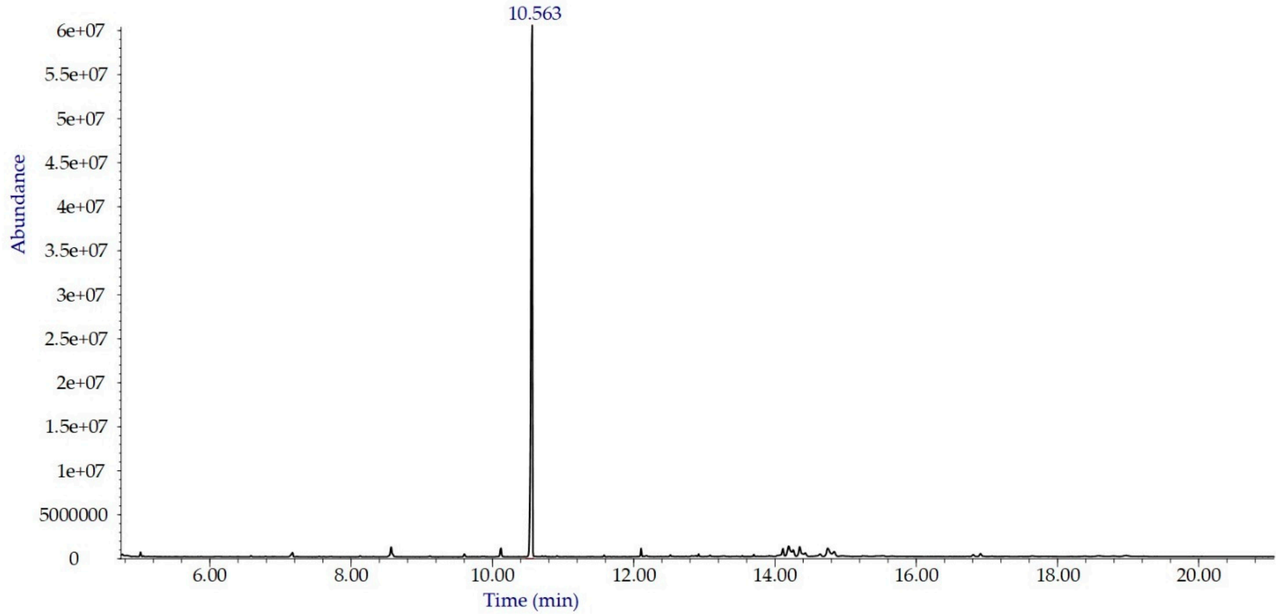

B)

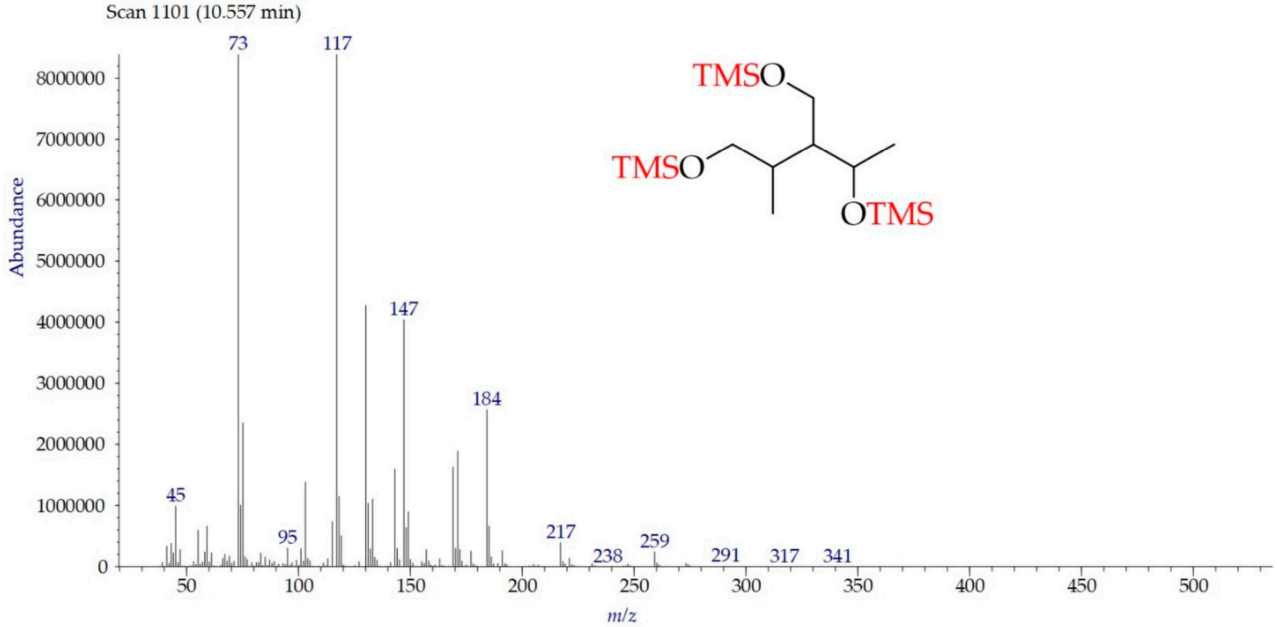

Figure S7. GC-MS analysis of purified chromatographic fraction containing nigrosphaeritriol (**1**) after trimethylsilylation with BSTFA: A) Total ion chromatogram and B) EI mass spectrum at 70 eV extracted at 10.557 min (RI = 1515). TMS = trimethylsilyl group.

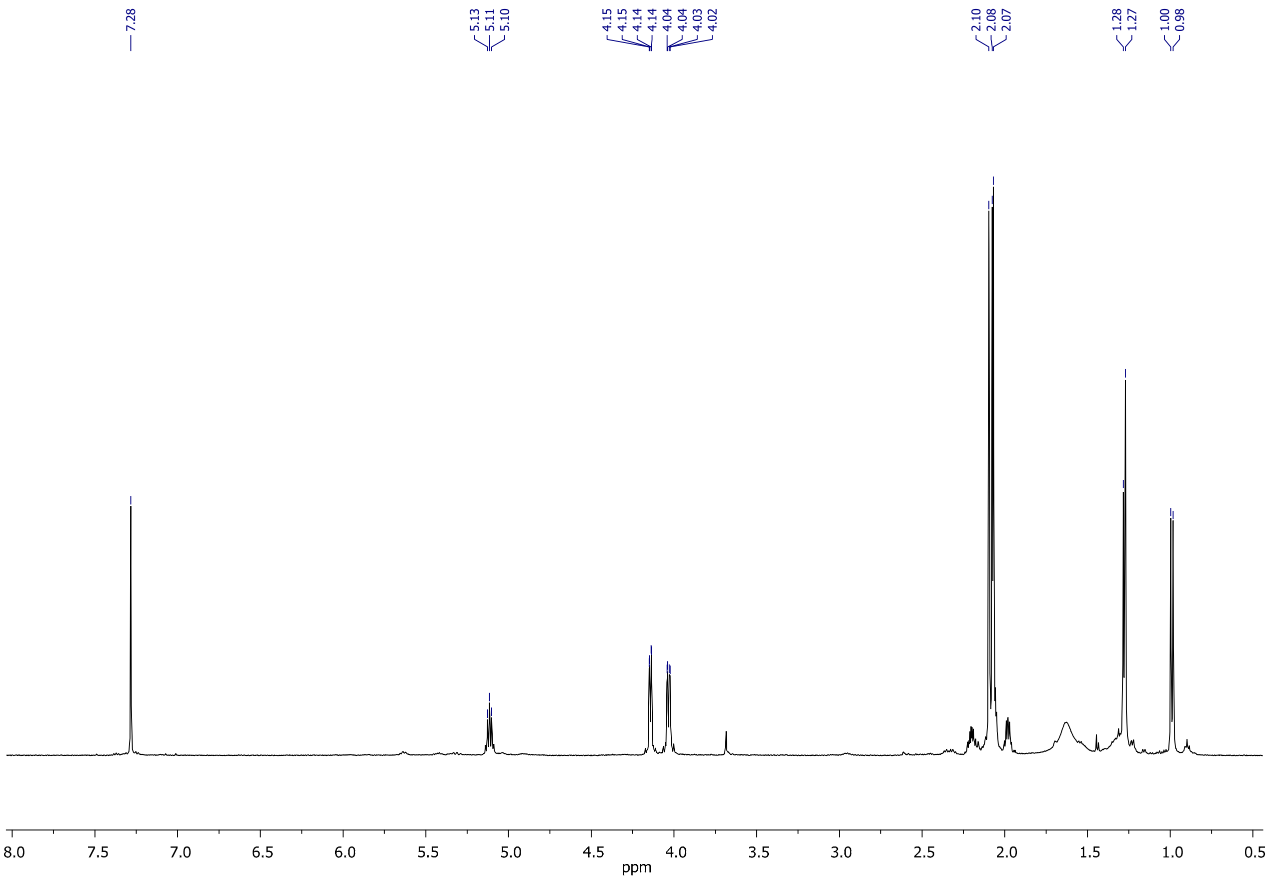

Figure S8. ^{1}H NMR spectrum of 1,1′,4-*O*,*O*′,*O*′′-triacetylnigrosphaerilactol (**17**), recorded at 400 MHz in $CDCl_3$.

A)

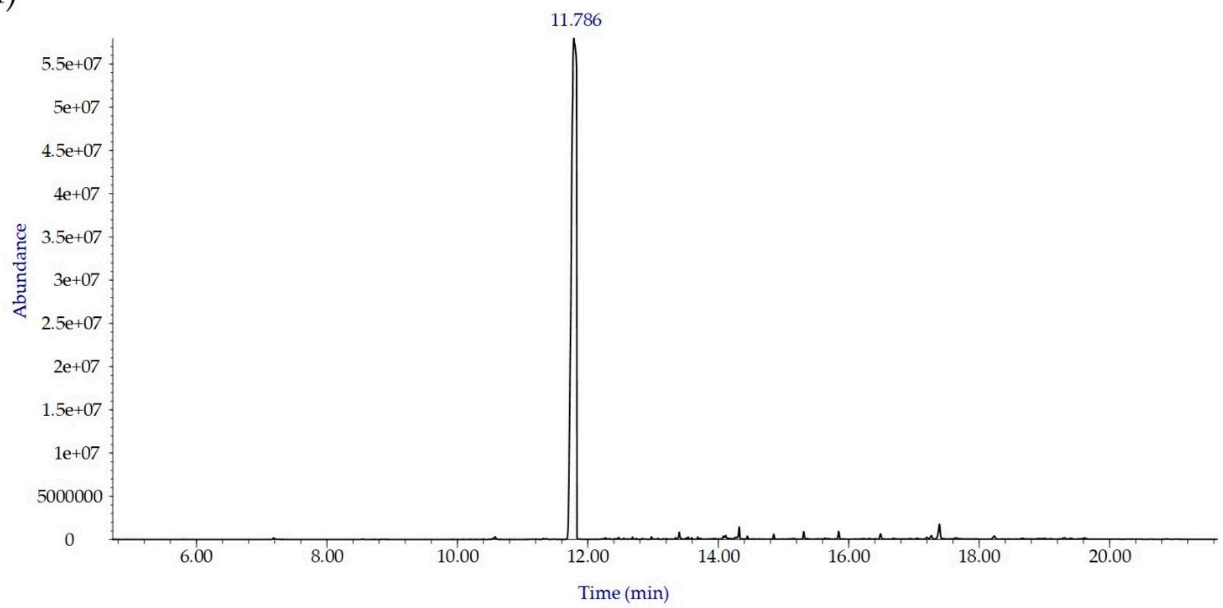

B)

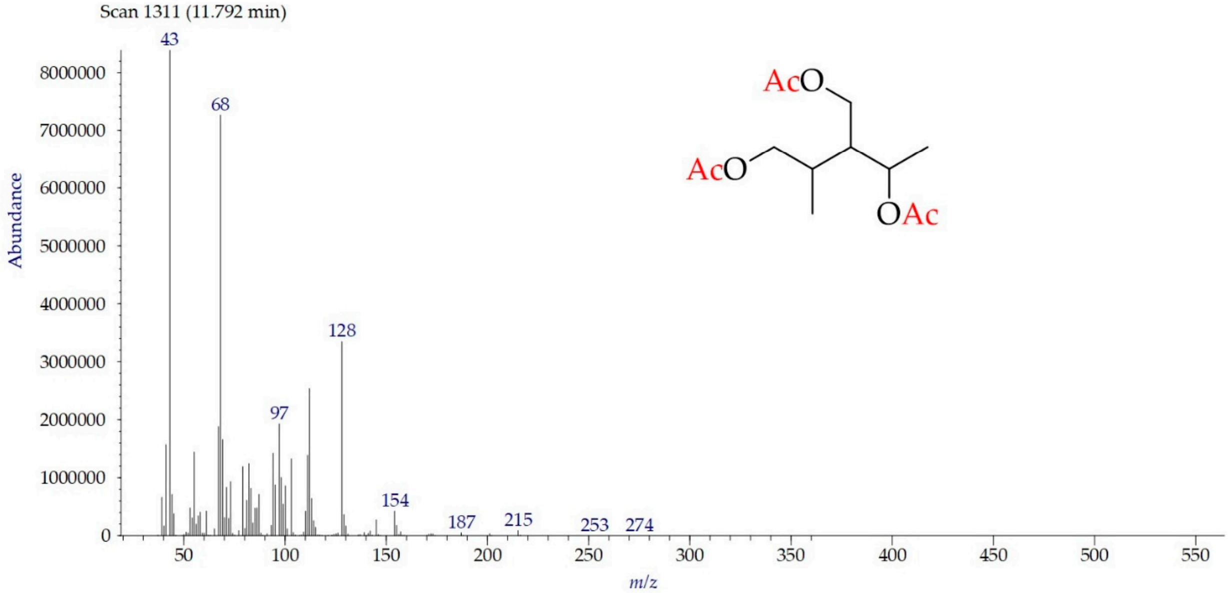

Figure S9. GC-MS analysis of purified chromatographic fraction containing nigrosphaeritriol (1) after acetylation (1,1′,4-*O*,*O*′,*O*′′-triacetylnigrosphaerilactol (**16**)): A) Total ion chromatogram and B) EI mass spectrum at 70 eV extracted at 11.786 min (RI = 1671). Ac = acetyl group.

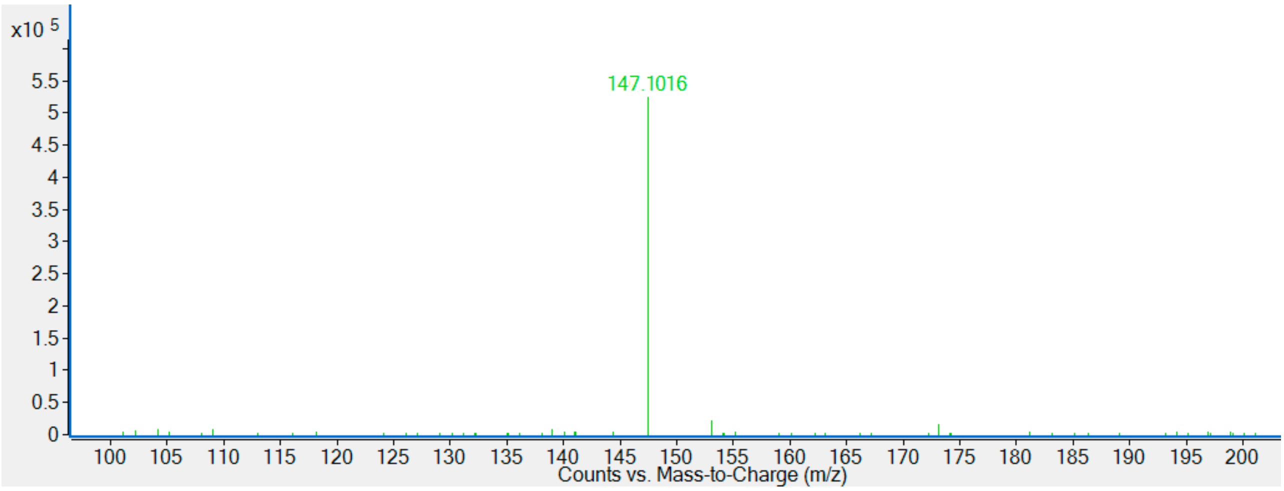

Figure S10. HR-ESI-MS spectrum of nigrosphaerilactol (**2**) recorded in positive modality.

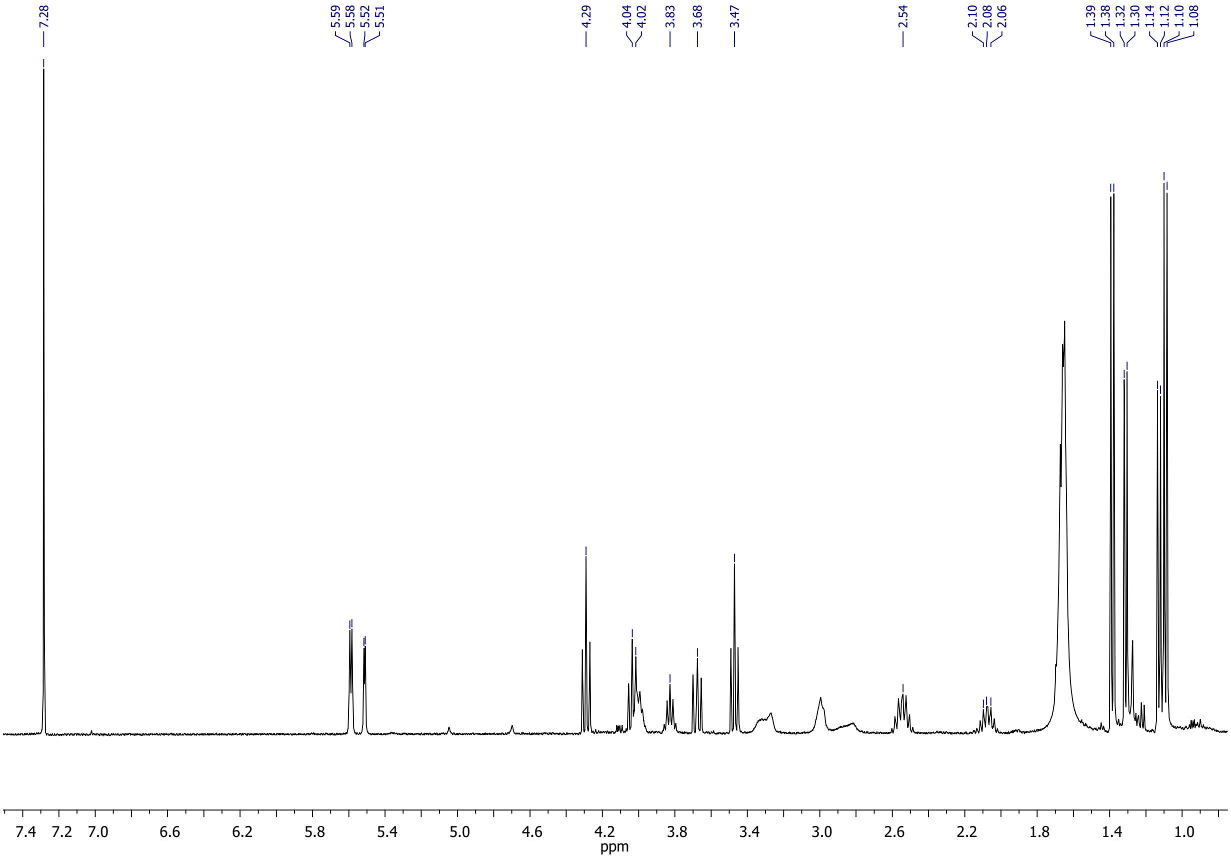

Figure S11. ^{1}H NMR spectrum of nigrosphaerilactol (**2**) recorded at 500 MHz in $CDCl_3$.

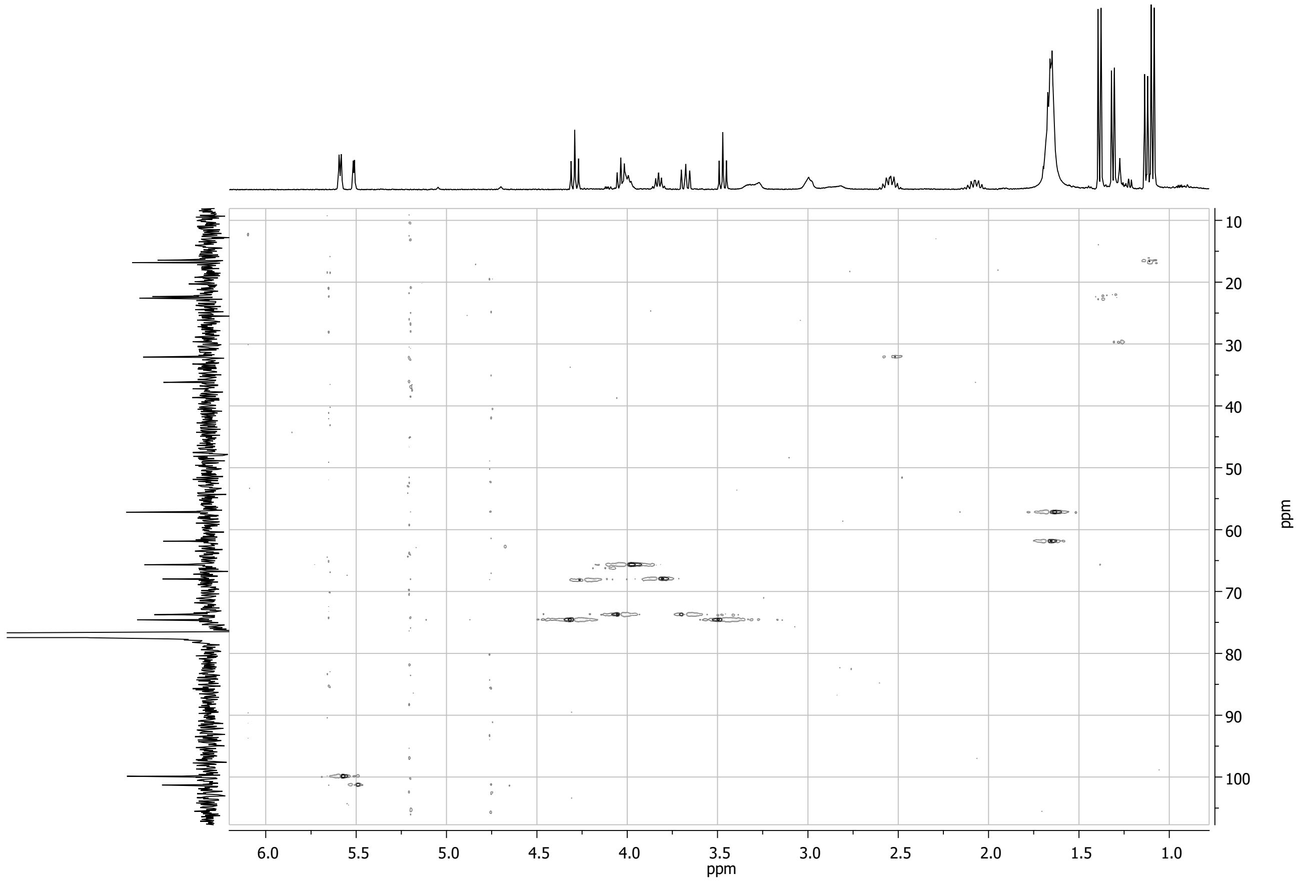

Figure S12. HSQC spectrum of nigrosphaerilactol (**2**) recorded at 500 MHz in $CDCl_3$.

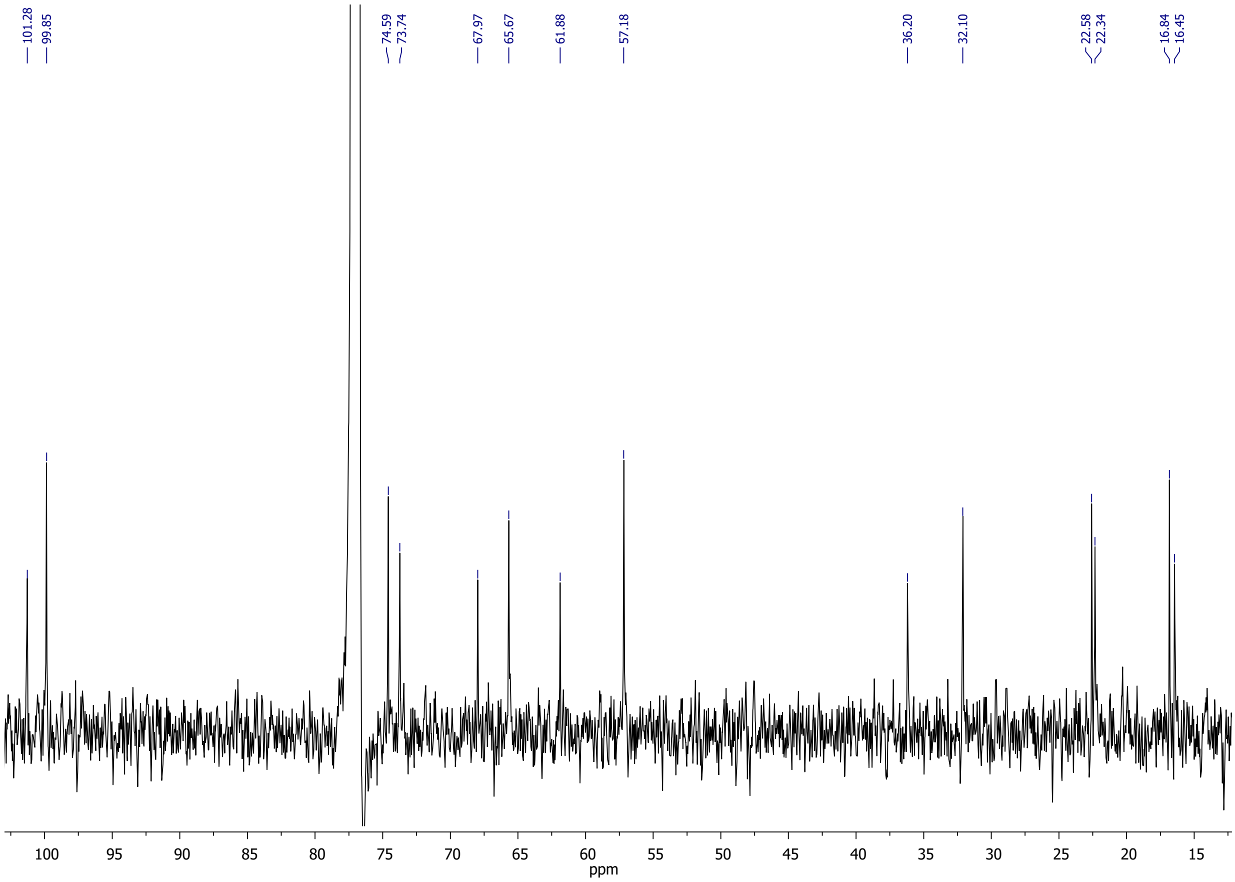

Figure S13. ^{13}C NMR spectrum of nigrosphaerilactol (**2**) recorded at 100 MHz in $CDCl_3$.

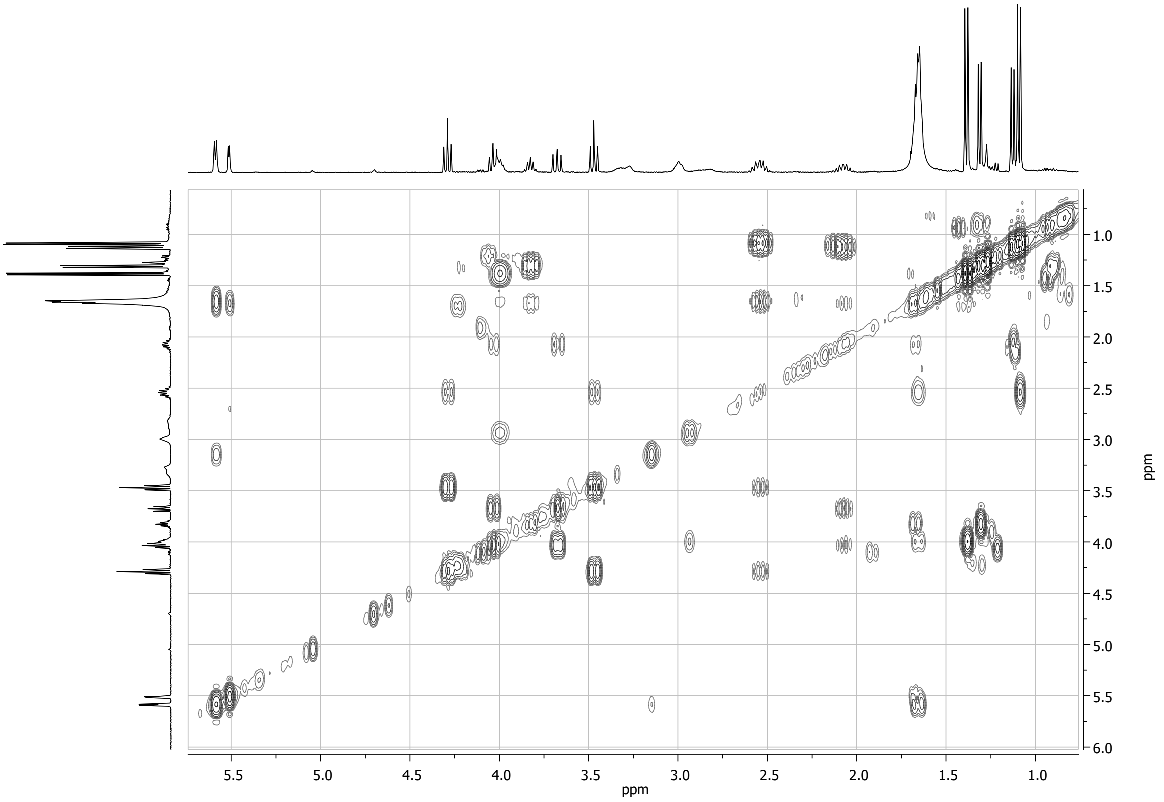

Figure S14. COSY spectrum of nigrosphaerilactol (**2**) recorded at 500 MHz in $CDCl_3$.

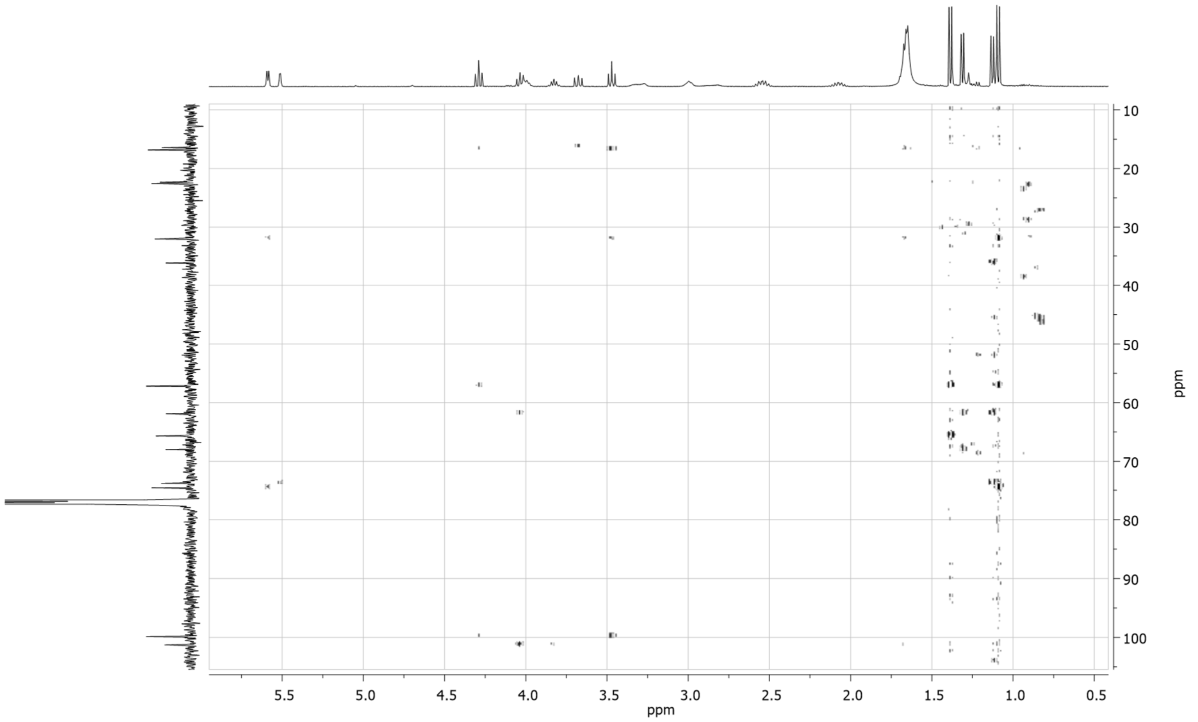

Figure S15. HMBC spectrum of nigrosphaerilactol (**2**) recorded at 500 MHz in $CDCl_3$.

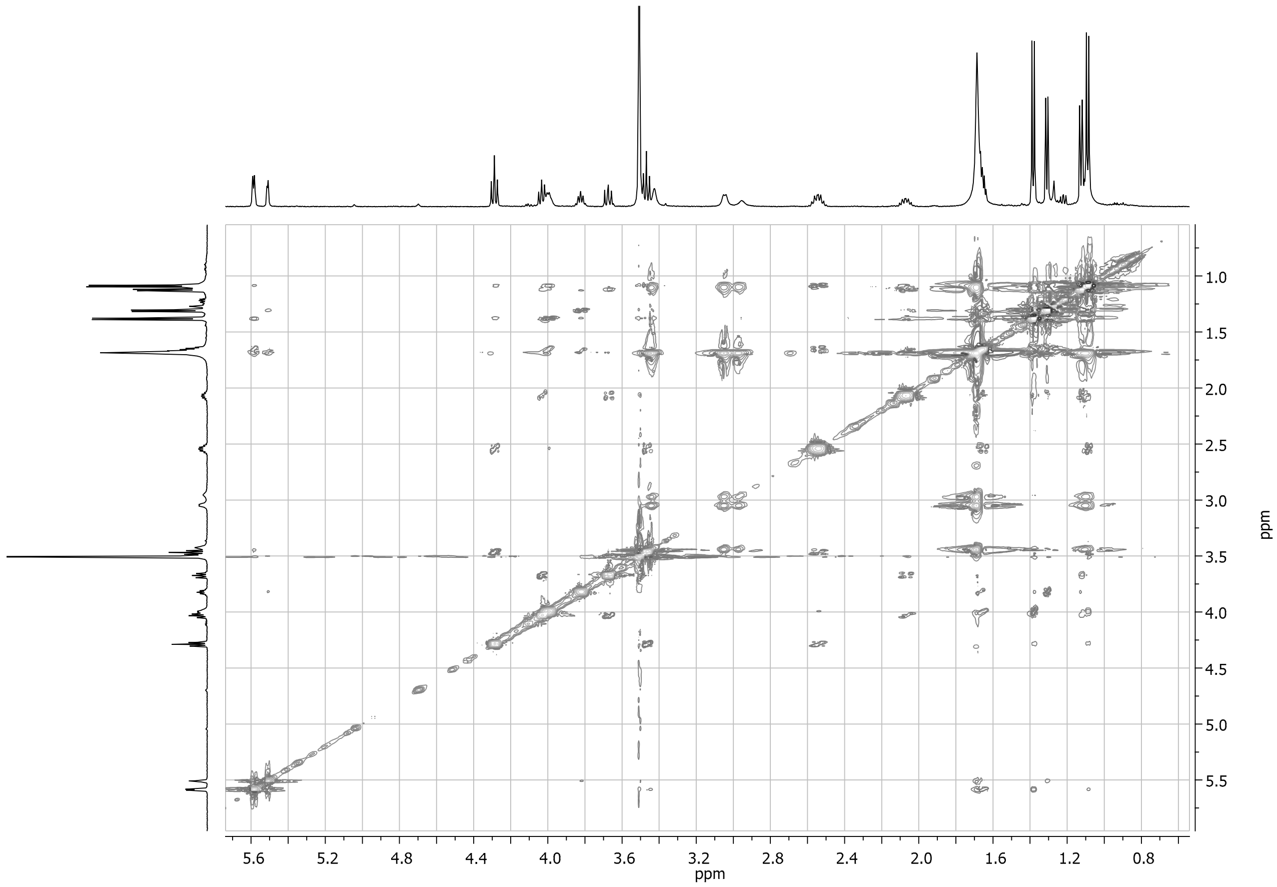

Figure S16. NOESY spectrum of nigrosphaerilactol (**2**) recorded at 500 MHz in $CDCl_3$.

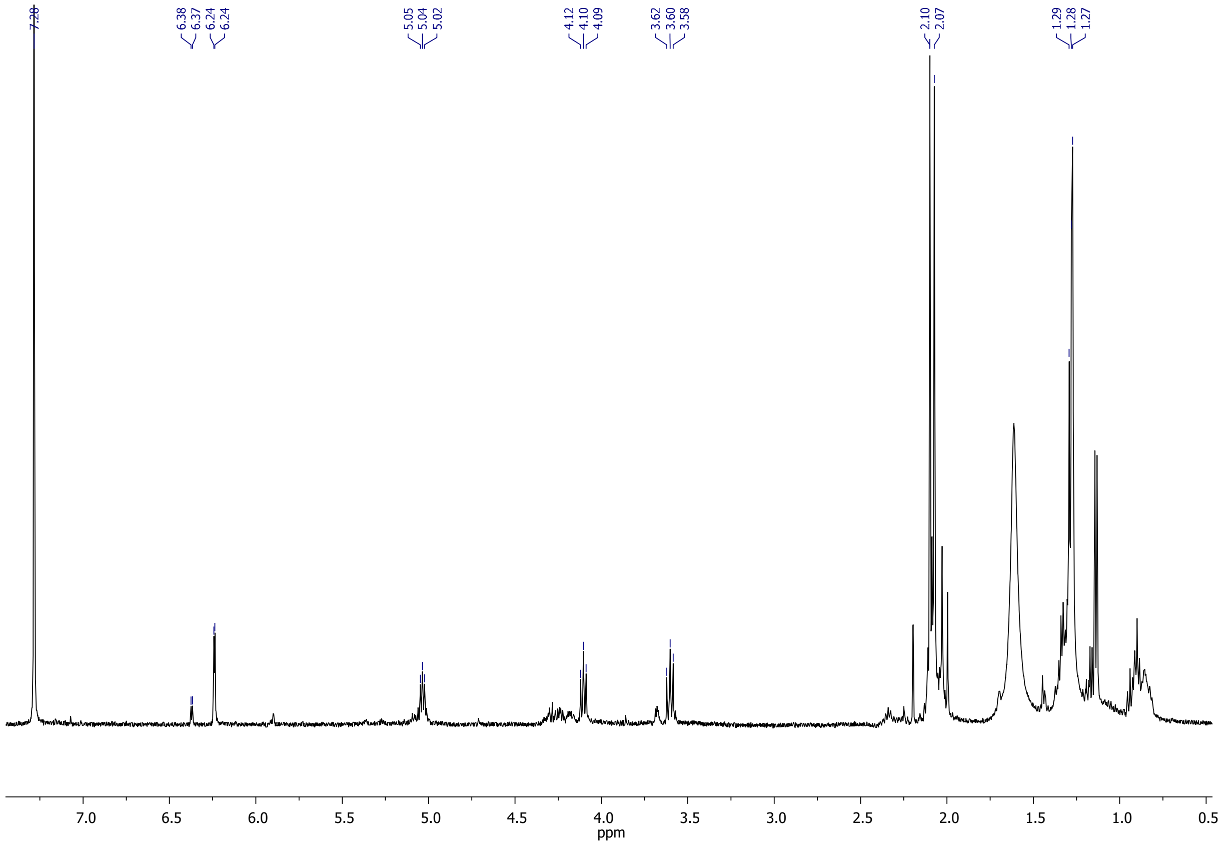

Figure S17. ^{1}H NMR spectrum of 2,6-*O,O*-diacetylnigrosphaerilactol (**17**) recorded at 400 MHz in $CDCl_3$.

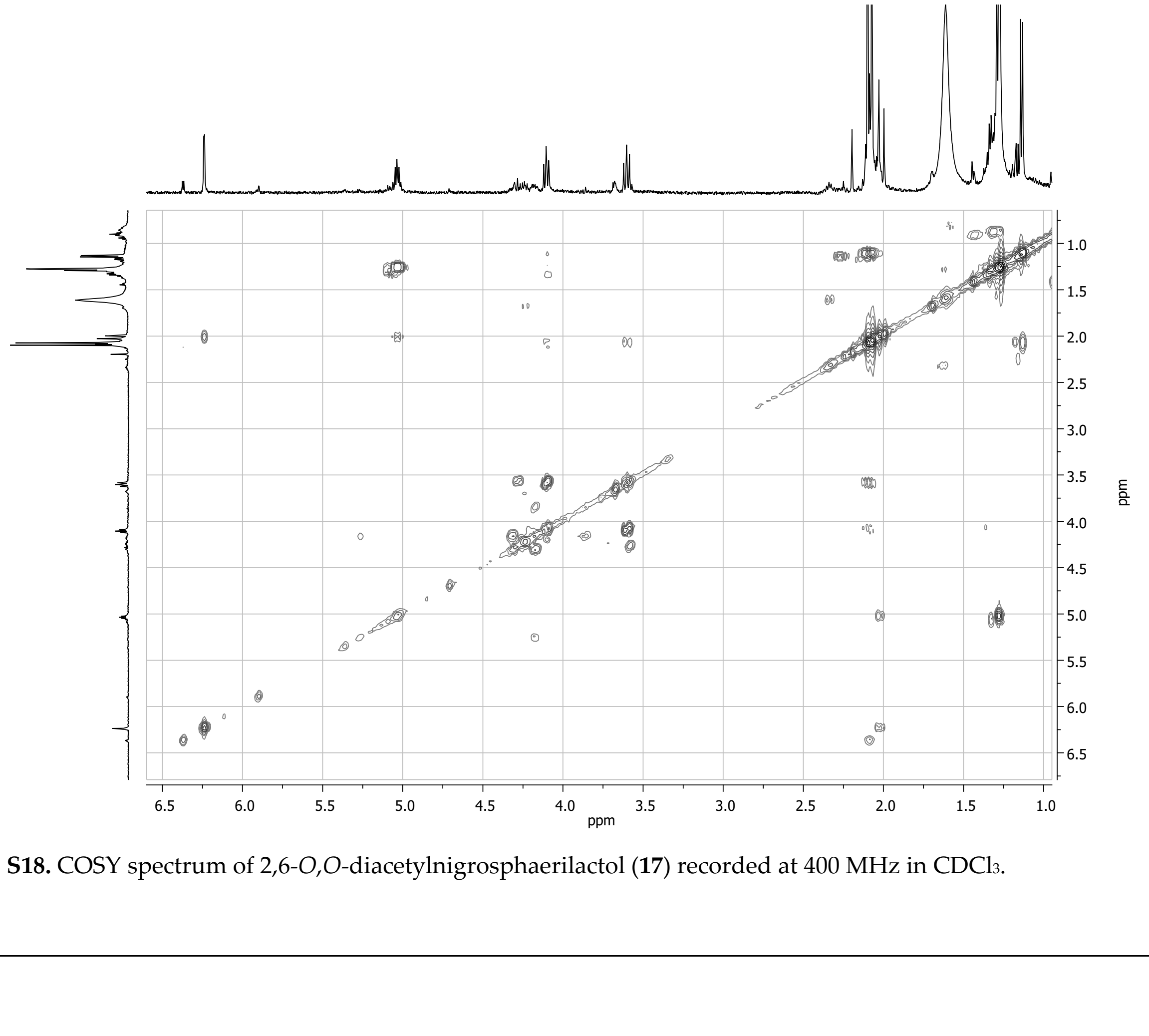

Figure S18. COSY spectrum of 2,6-*O,O*-diacetylnigrosphaerilactol (**17**) recorded at 400 MHz in $CDCl_3$.

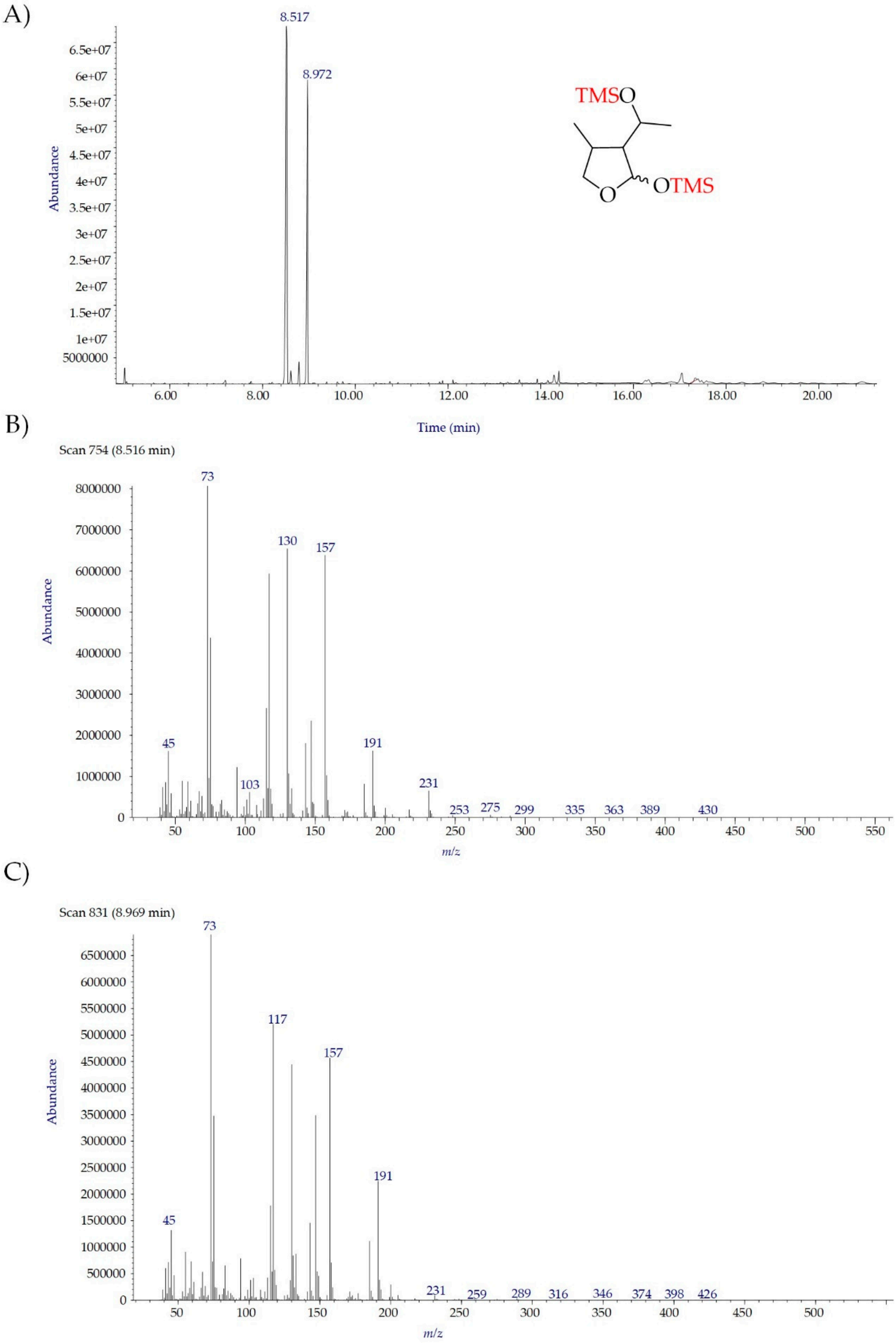

Figure S19. GC-MS analysis of purified chromatographic fraction containing nigrosphaerilactol (**2**) after trimethylsilylation with BSTFA: A) Total ion chromatogram; B) EI mass spectrum at 70 eV extracted at 8.516 min (RI =1346); C) EI mass spectrum at 70 eV extracted at 8.969 min (RI = 1381). TMS = trimethylsilyl group.

A)

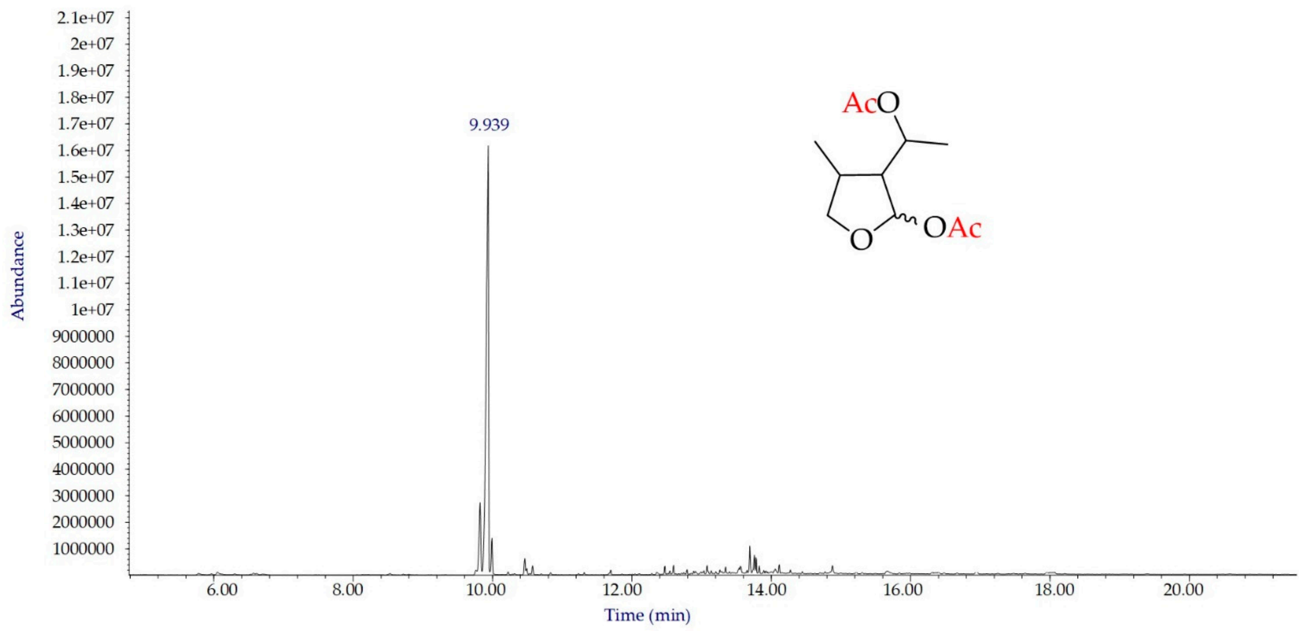

B)

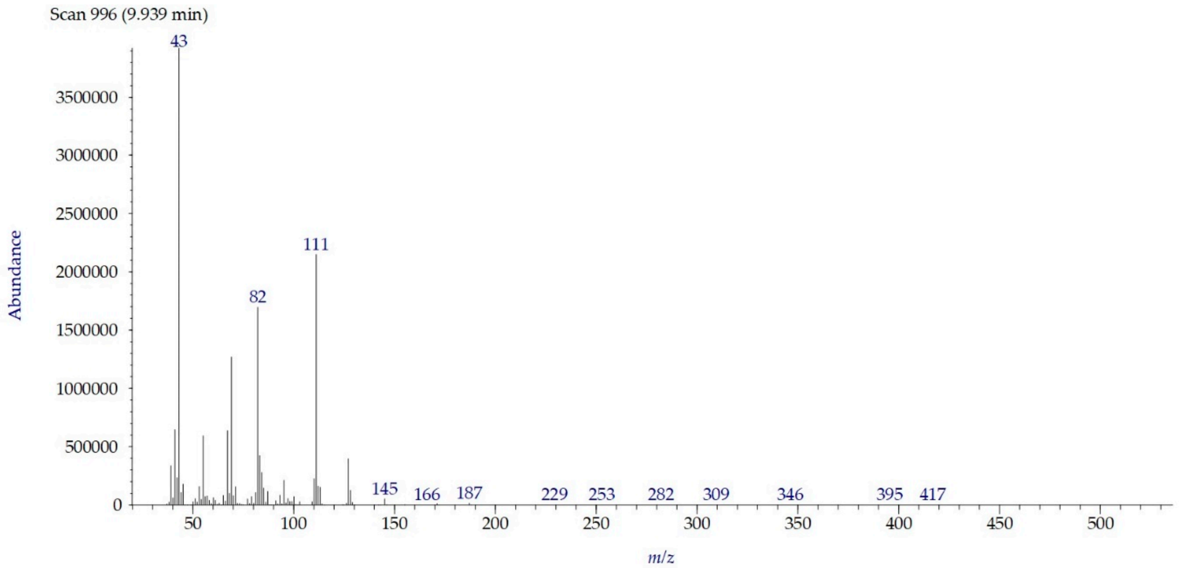

Figure S20. GC-MS analysis of purified chromatographic fraction containing nigrosphaerilactol (**2**) after acetylation (2,6-*O*,*O'*-diacetylnigrospaerilactol, **17**): A) Total ion chromatogram and B) EI mass spectrum at 70 eV extracted at 9.939 min (RI = 1459). Ac = acetyl group.

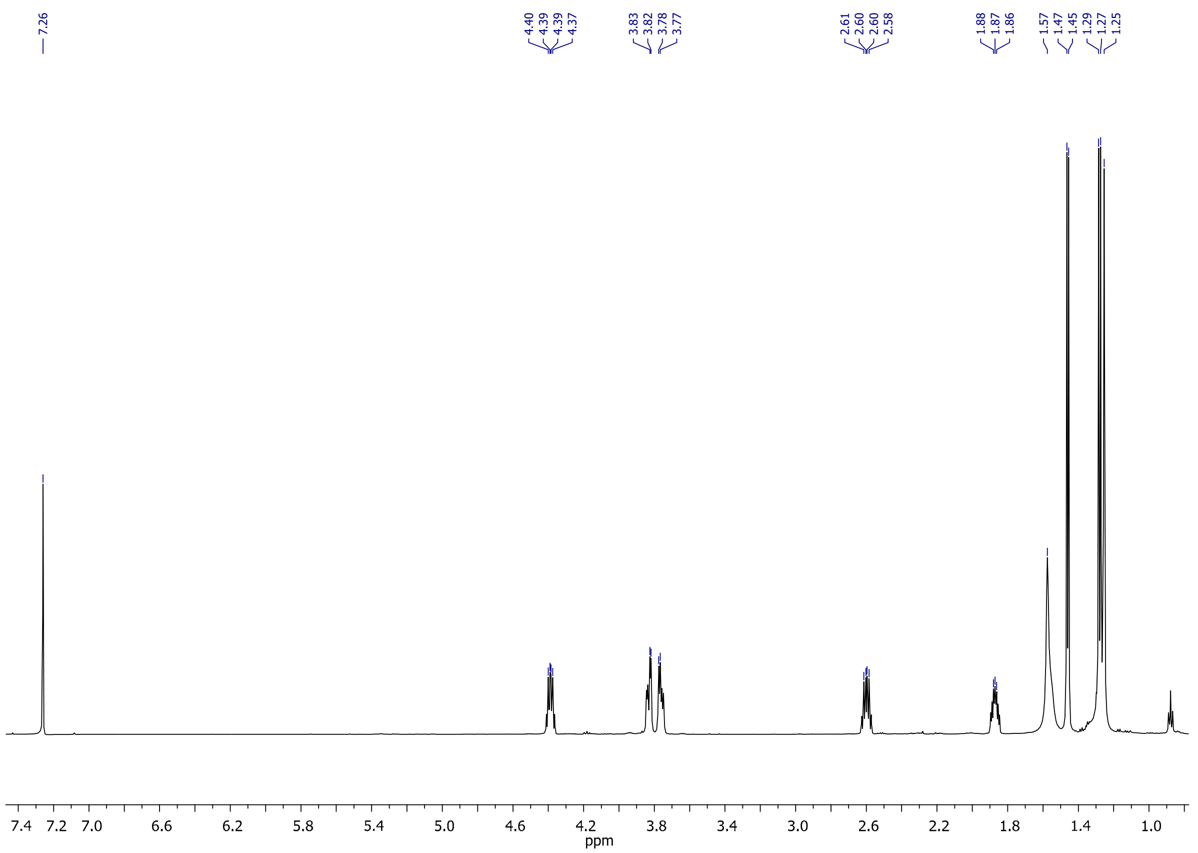

Figure S21. ^{1}H NMR spectrum of nigrosphaerilactone (**3**) recorded at 400 MHz in $CDCl_3$.

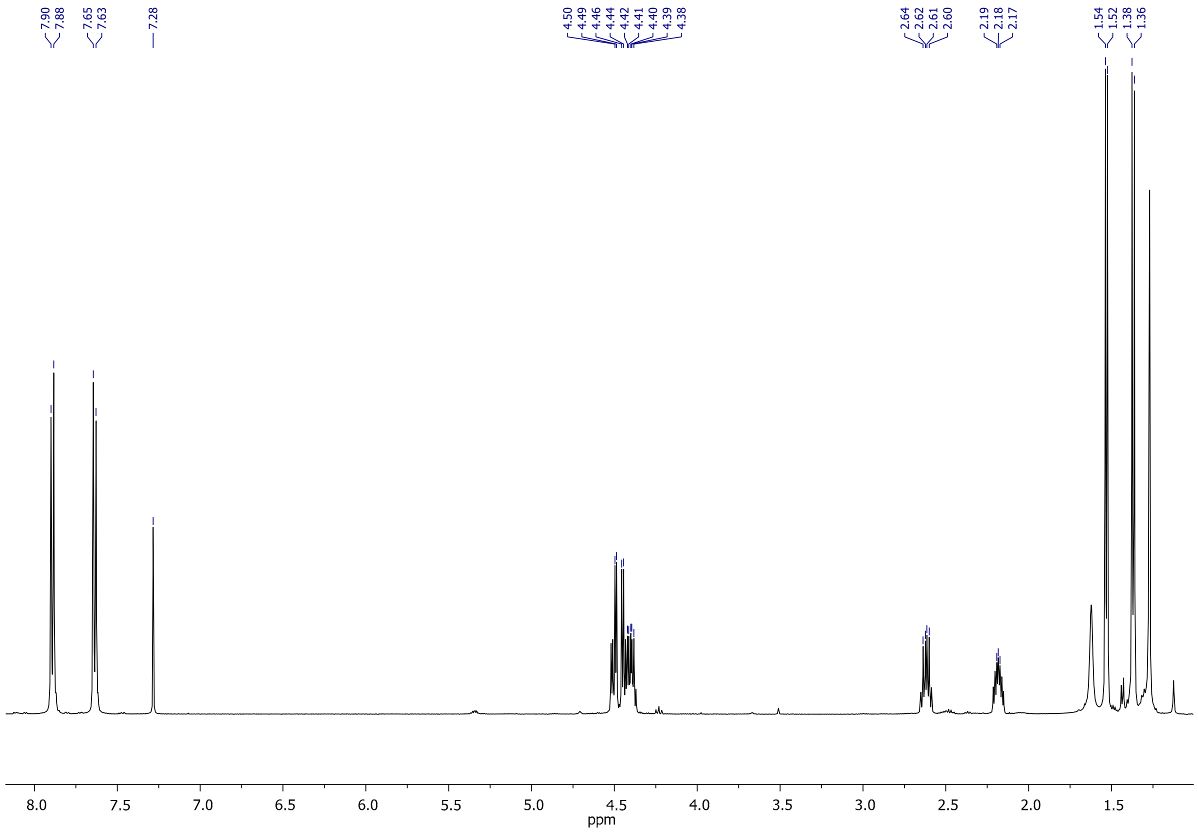

Figure S22. ^{1}H NMR spectrum of 8-*O*-*p*-bromobenzoylnigrosphaerilactone (**18**) recorded at 400 MHz in $CDCl_3$.

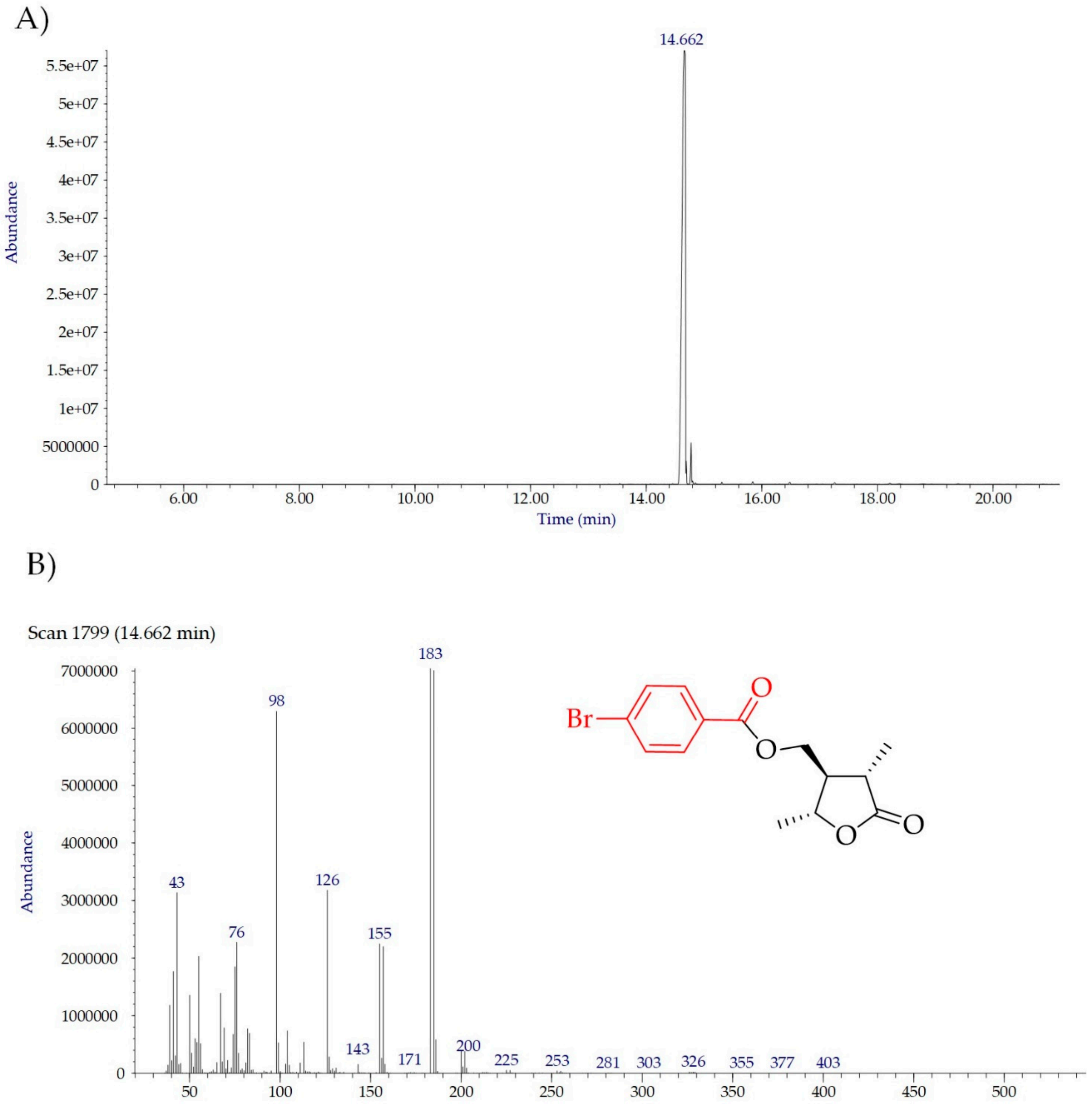

Figure S23. GC-MS analysis of purified chromatographic fraction containing nigrosphaerilactone (**2**) with *p*-bromobenzoyl chloride giving *8-O-p*-bromobenzoynigrosphaerilactone (**18**): A) Total ion chromatogram and B) EI mass spectrum at 70 eV extracted at 14.662 min (RI = 2348).

Table S1. Crystal data and structure refinement for 8-*O*-*p*-bromobenzoylnigrosphaerilactone (**18**). Crystallographic data for the structure have also been deposited with the Cambridge Crystallographic Data Centre as supplementary publication number CCDC 2322188.

Empirical formula	$C_{14} H_{15} Br O_4$
Formula weight	327.17
Temperature	298(2) K
Wavelength	0.71073 Å
Crystal system, space group	Orthorhombic, P 21 21 21
Unit cell dimensions	a = 7.3450(6)Å α = 90°
	b = 11.486(2)Å β = 90°

	c = 16.5930(19)Å γ = 90°
Volume	1399.9(3)Å^3
Z, Calculated density	4, 1.552 Mg/m^3
Absorption coefficient	2.943mm^{-1}
F(000)	664
Crystal size	0.350 x 0.250 x 0.200mm
Theta range for data collection	3.029 to 27.503°
Limiting indices	-9<=h<=7, -14<=k<=14, -19<=l<=21
Reflections collected / unique	7875 / 3100 [R(int) = 0.0716]
Completeness to theta	=25.186 to 99.2%
Refinement method	Full-matrix least-squares on F^2
Data / restraints / parameters	3100 / 0 / 174
Goodness-of-fit on F^2	1.070
Final R indices [I>2sigma(I)]	R1 = 0.0465, wR2 = 0.0721
R indices (all data)	R1 = 0.1170, wR2 = 0.0933
Absolute structure parameter	0.071(13)
Extinction coefficient	n/a
Largest diff. peak and hole	0.369 and -0.519 e Å^{-3}

Table S2. Selected bond lengths and angles with standard deviations for 8-*O*-*p*-bromobenzoylnigrosphaerilactone (**18**).

O(1)-C(2)	1.341(9)
C(1)-C(5)	1.468(8)
O(2)-C(2)	1.199(9)
O(3)-C(8)	1.441(7)
O(3)-C(9)	1.336(7)
O(4)-C(9)	1.204(7)
Br(1)-C(13)	1.880(7)
C(4)-C(8)-O(3)-C(9)	167.2(5)
C(8)-O(3)-C(9)-C(10)	-178.4(5)
O(3)-C(9)-C(10)-C(11)	176.3(7)

Table S3. Hydrogen bonds for 8-*O*-*p*-bromobenzoylnigrosphaerilactone (**18**).

D-H...A	d(D-H)	d(H...A)	d(D...A)	<(DHA)
C(12)-H(12)...O(1)#1	0.93	2.53	3.324(8)	143.9
C(6)-H(6B)...O(3)	0.96	2.65	3.223(8)	118.4

Symmetry transformations used to generate equivalent atoms: #1 x,y+1,z

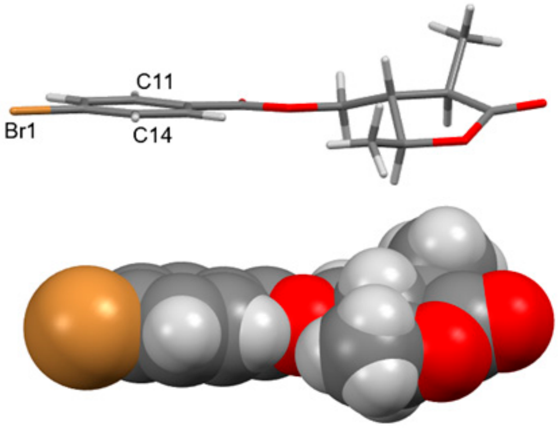

Figure S24. Perspective view of the molecule in the edge of phenyl plane in the Capped Sticks style (up) and Spacefill style (down) showing flat elongated shape of the molecule.

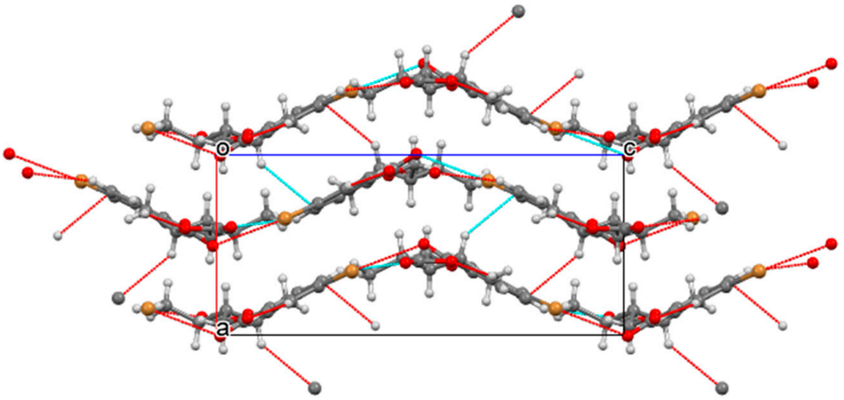

Figure S25. Crystal packing viewed along **b** axis showing waved sheets of molecules. Contacts shorter than the sum of vdW radii are drawn as cyan and red dashed lines.

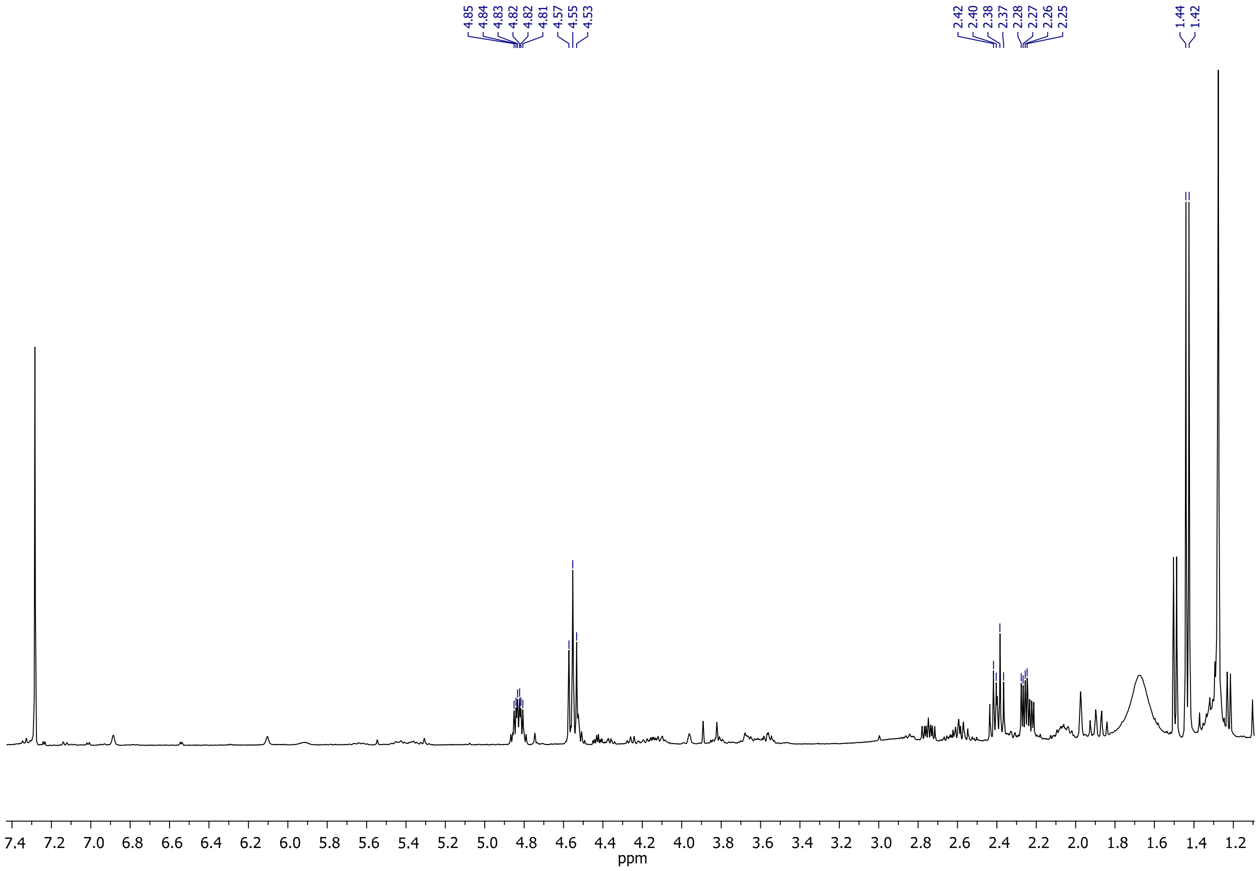

Figure S26. ^{1}H NMR spectrum of (*S*)-hydroxybutyrolactone (**4**) recorded at 400 MHz in $CDCl_3$.

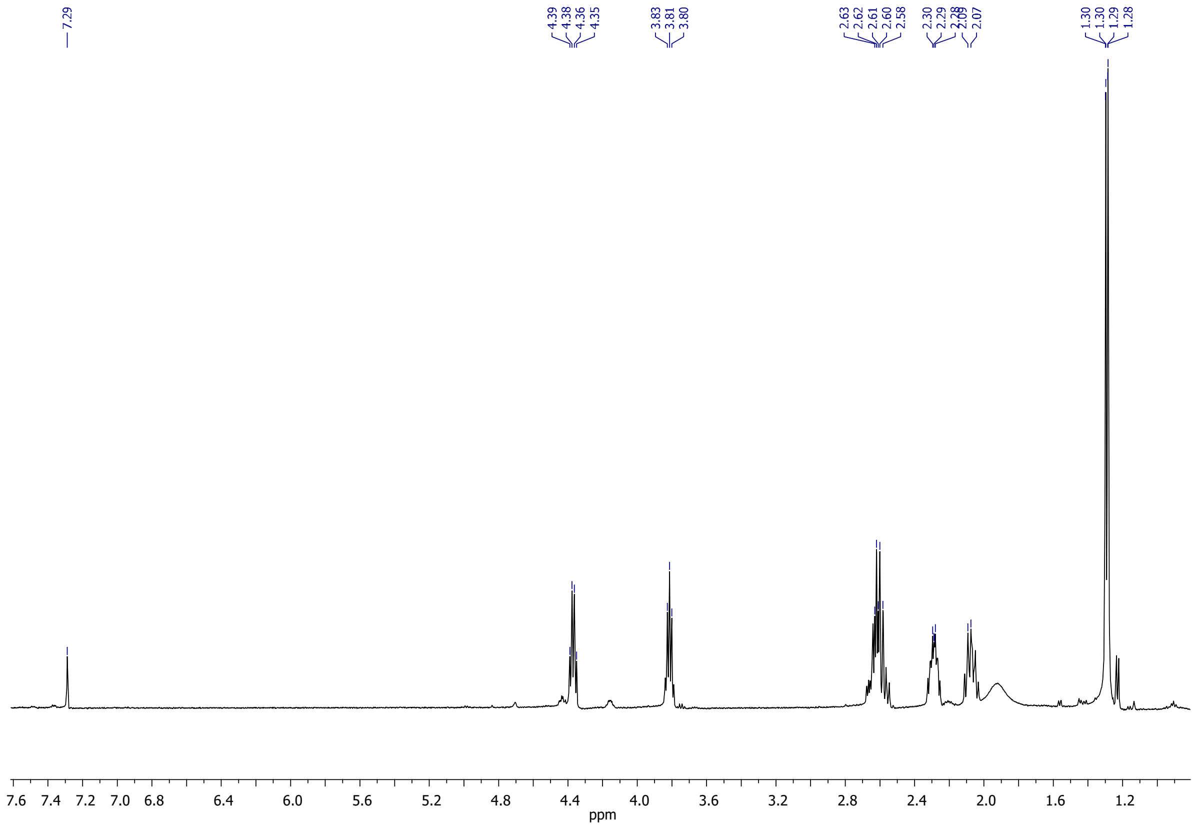

Figure S27. ^{1}H NMR spectrum of lupinlactone (**5**) recorded at 400 MHz in $CDCl_3$.

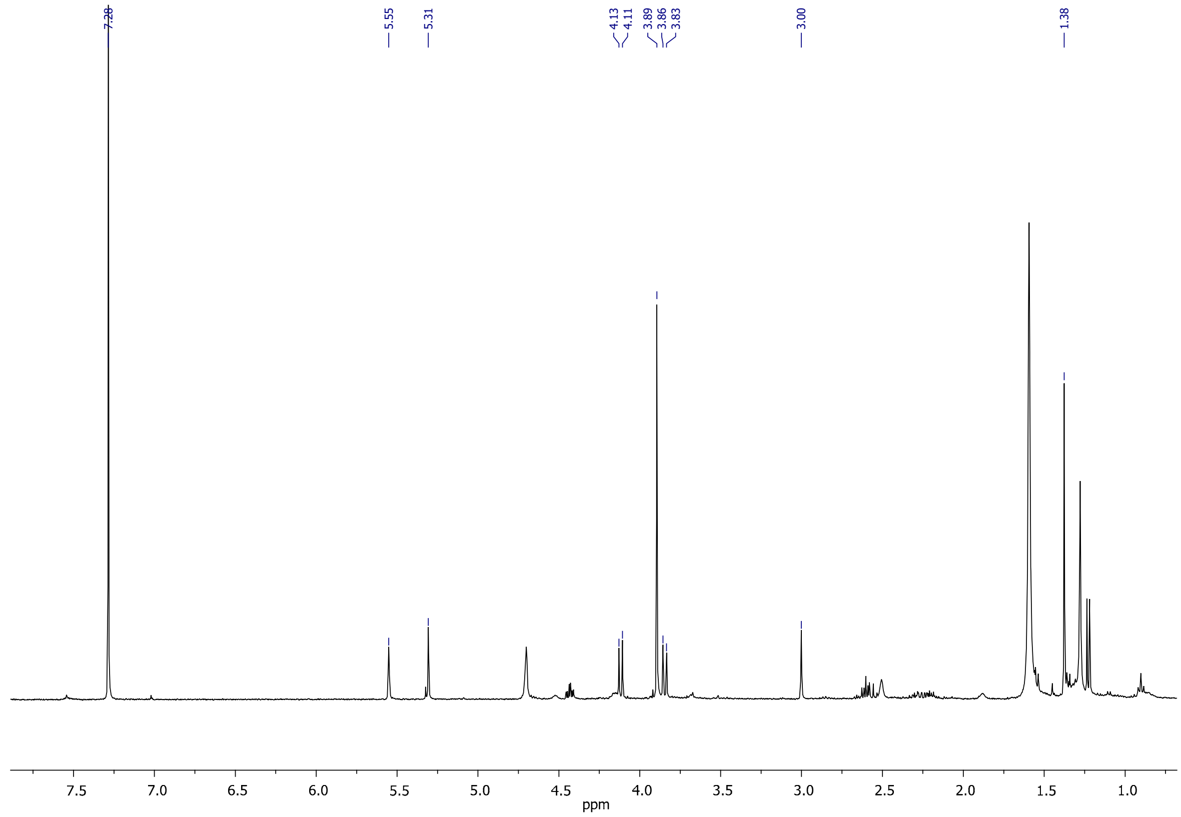

Figure S28. ^1H NMR spectrum of nigrosporione A (**6**) recorded at 400 MHz in $CDCl_3$.

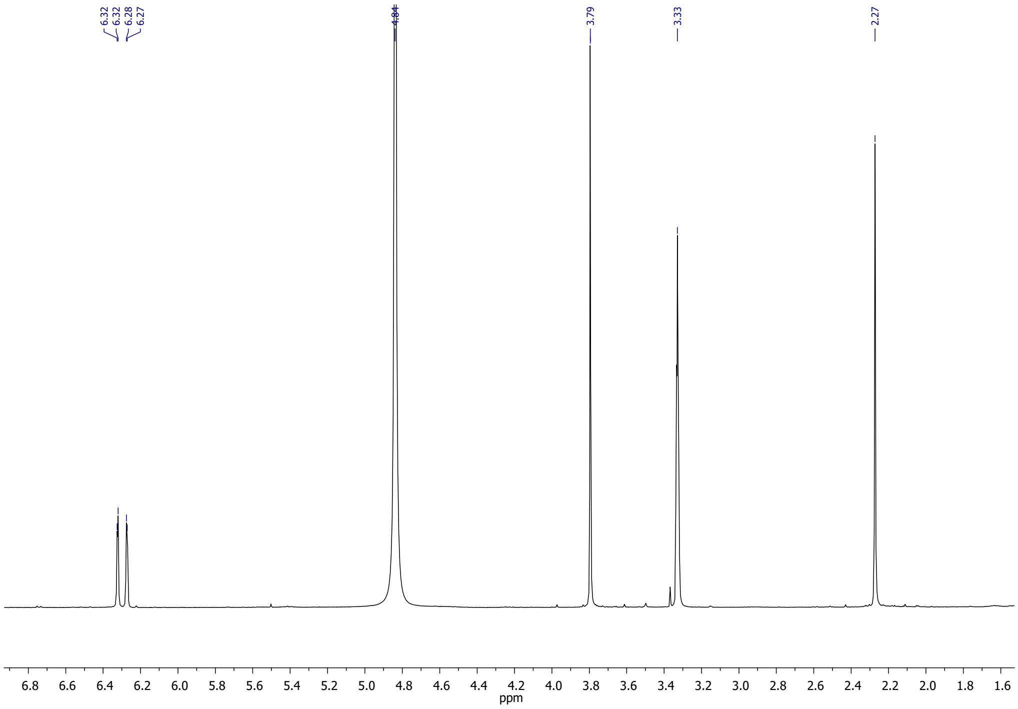

Figure S29. ^1H NMR spectrum of 2,4-dihydroxy-6-methoxyacetophenone (**7**) recorded at 400 MHz in CD_3OD.

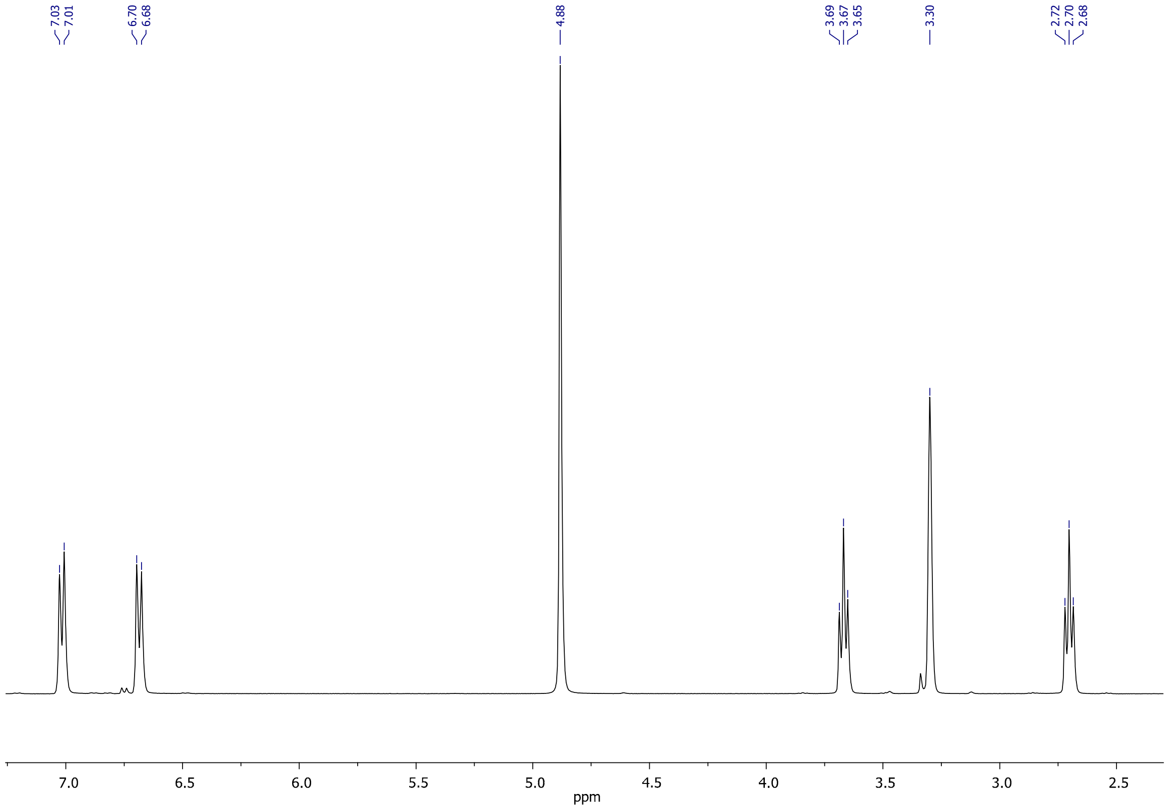

Figure S30. ^1H NMR spectrum of tyrosol (**8**) recorded at 400 MHz in CD_3OD.

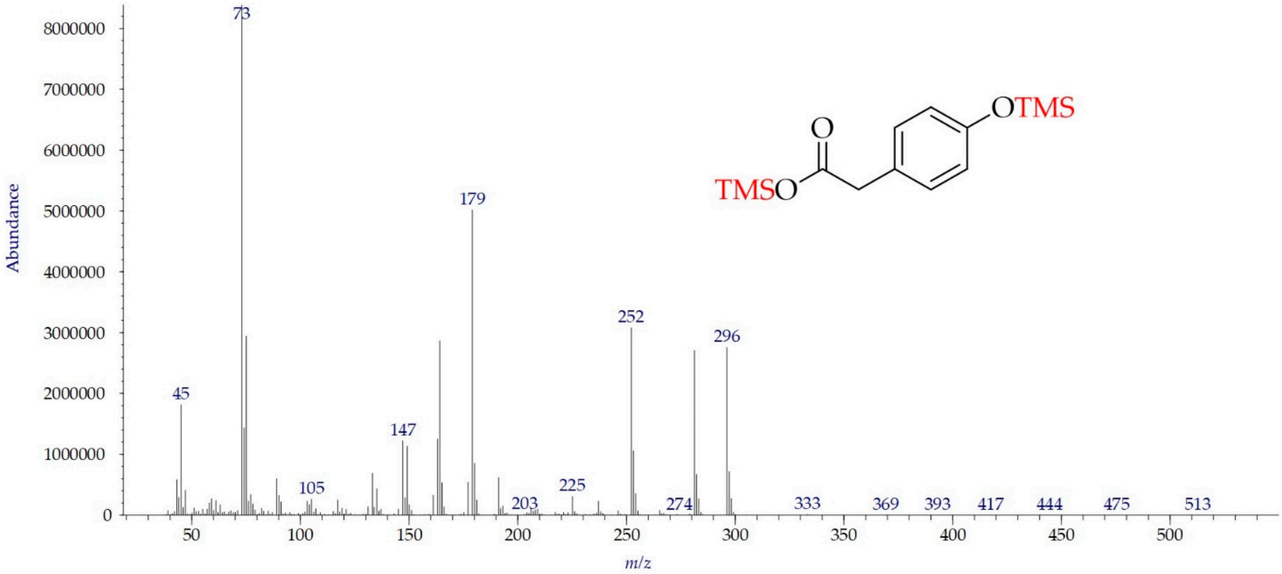

Figure S31. EI mass spectrum at 70 eV of 4-hydroxyphenylacetic acid (**9**), 2TMS (RI = 1581). TMS = trimethylsilyl group.

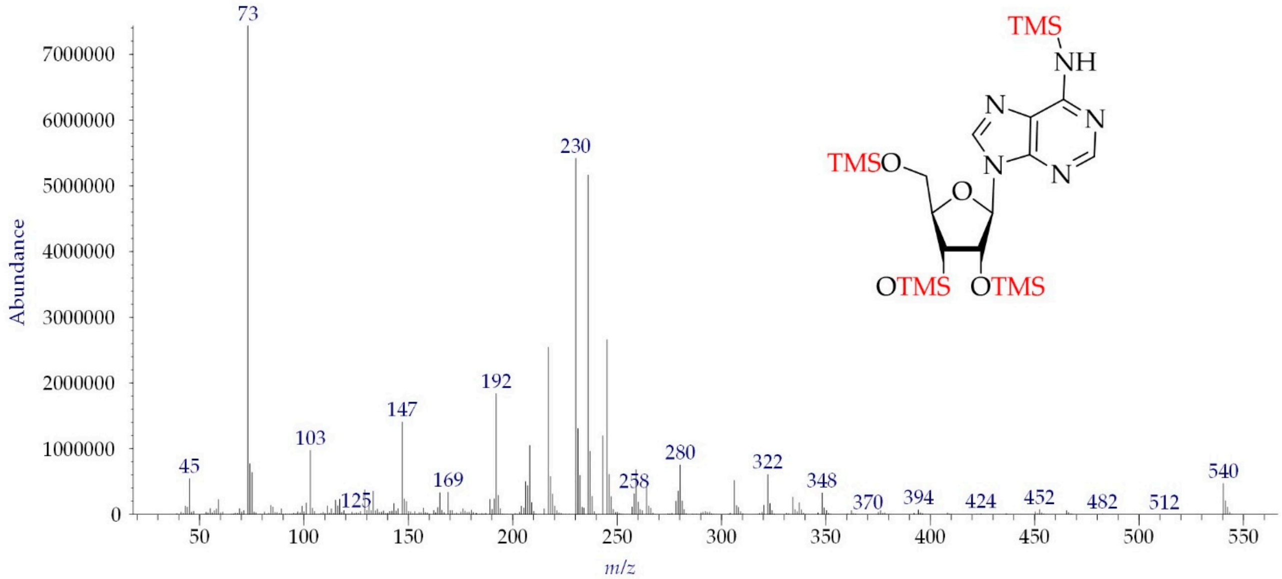

Figure S32. EI mass spectrum at 70 eV of adenosine (**10**), 4TMS (RI = 2670). TMS = trimethylsilyl group.

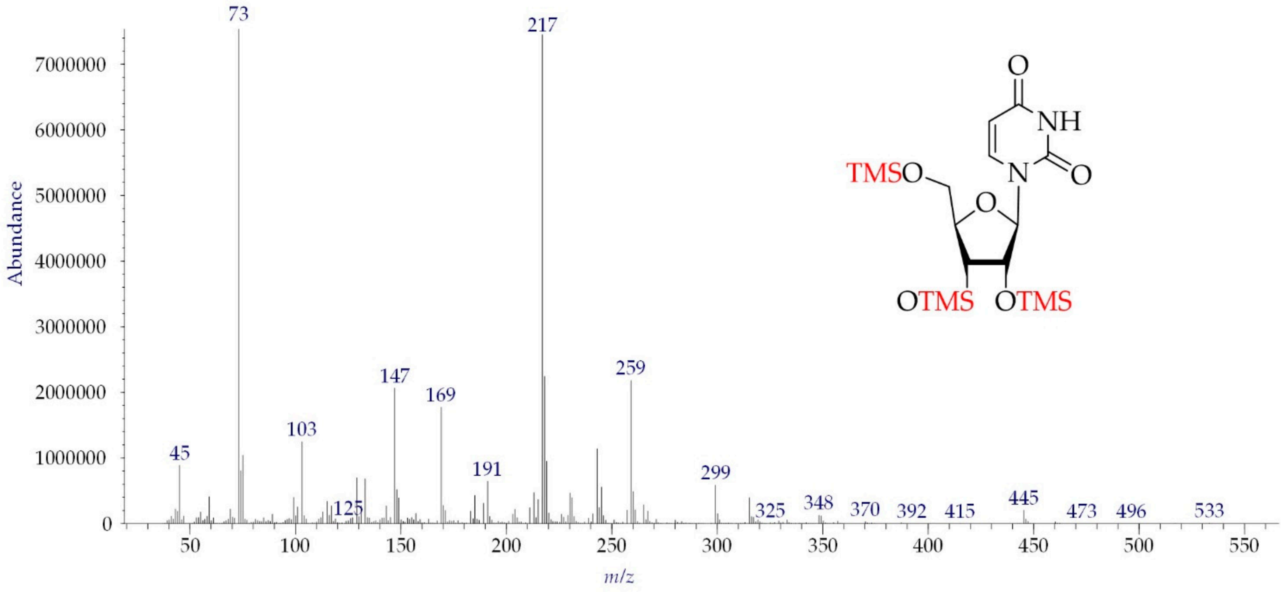

Figure S33. EI mass spectrum at 70 eV of uridine (**11**), 3TMS (RI = 2496). TMS = trimethylsilyl group.

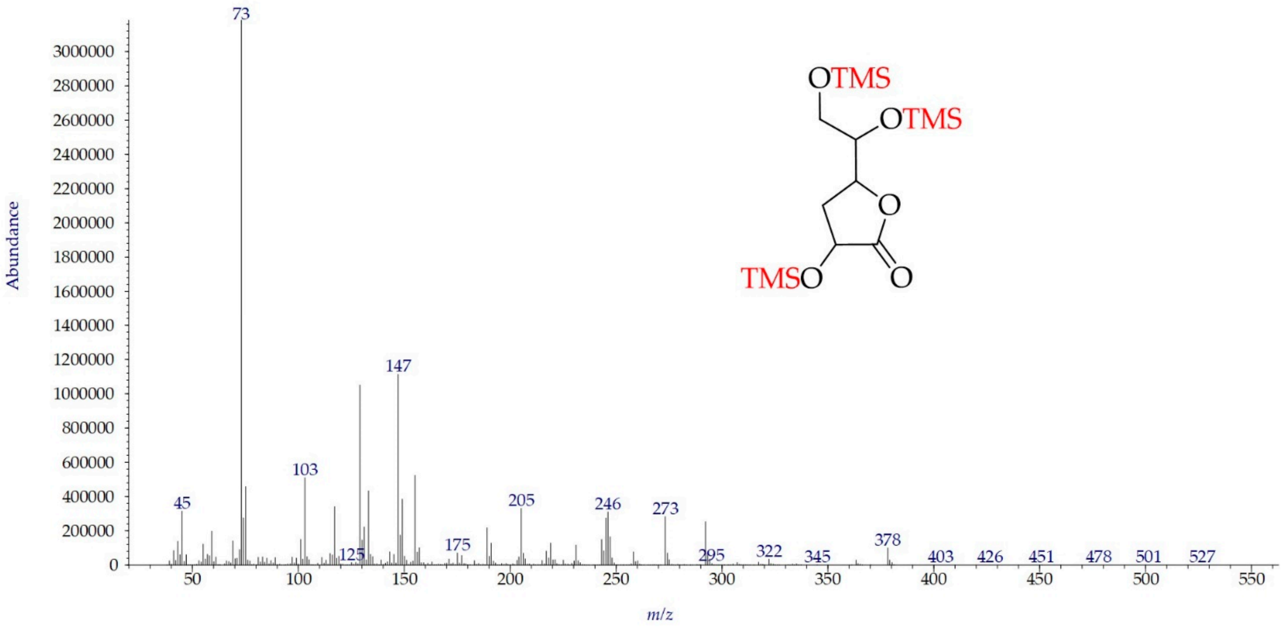

Figure S34. EI mass spectrum at 70 eV of ribo-hexonic acid 3-deoxy-γ-lactone (**12**), 3TMS (RI = 1795). TMS = trimethylsilyl group.

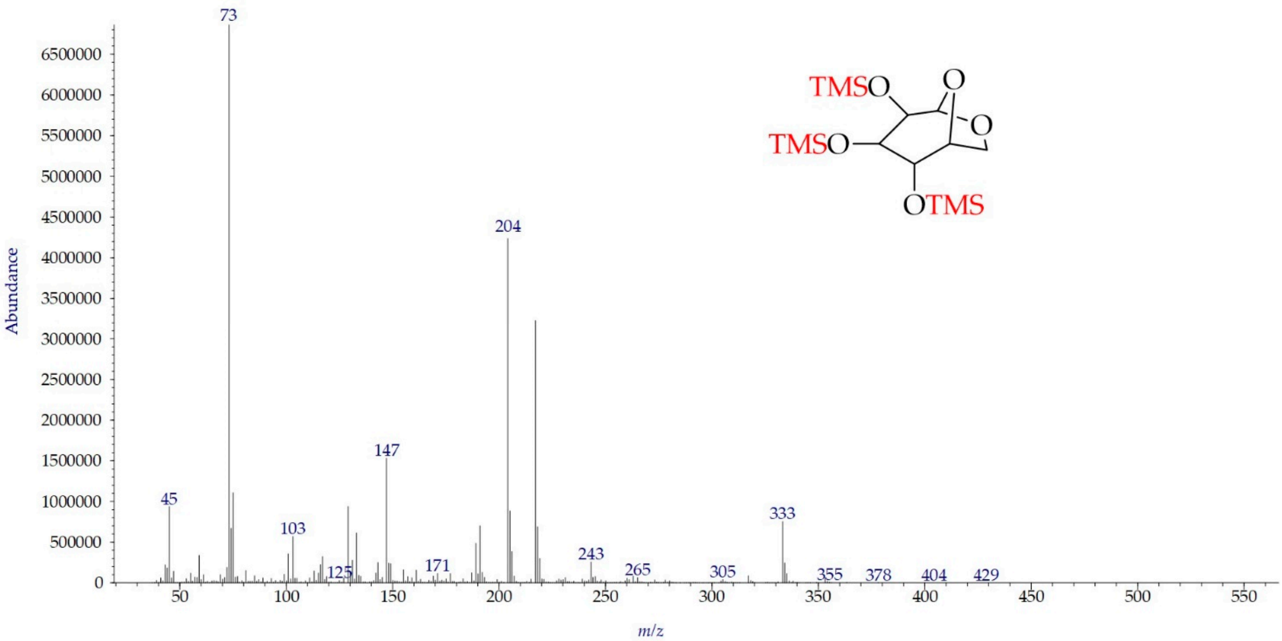

Figure S35. EI mass spectrum at 70 eV of levoglucosan (**13**), 3TMS (RI = 1726). TMS = trimethylsilyl group.

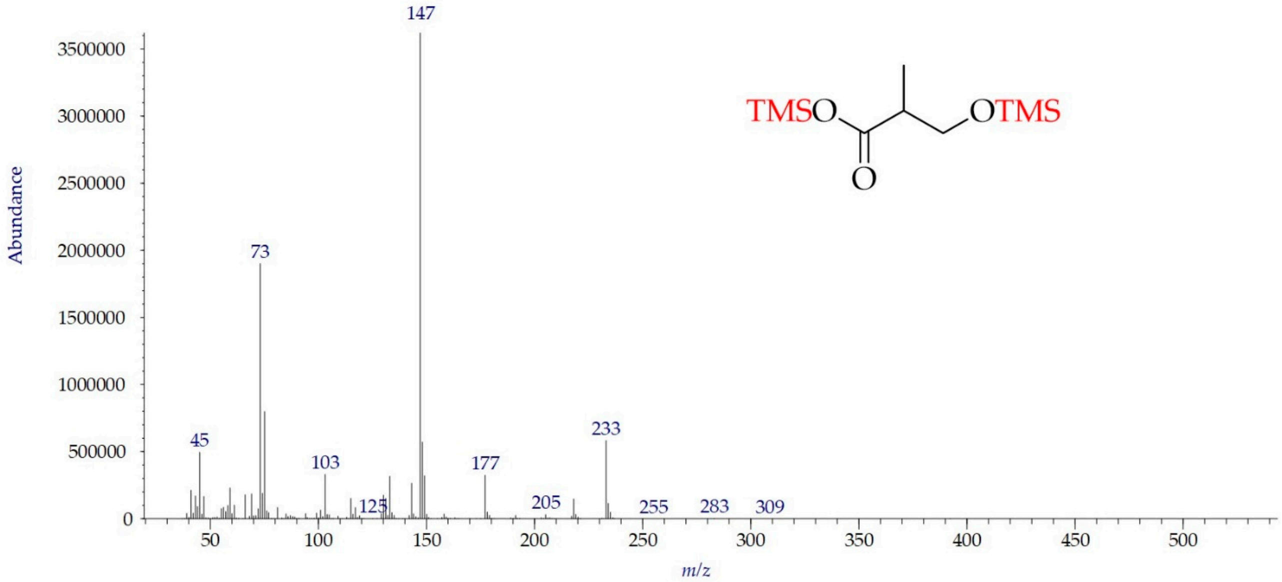

Figure S36. EI mass spectrum at 70 eV of 3-hydroxyisobutyric acid (**14**), 2TMS (RI = 1167). TMS = trimethylsilyl group.

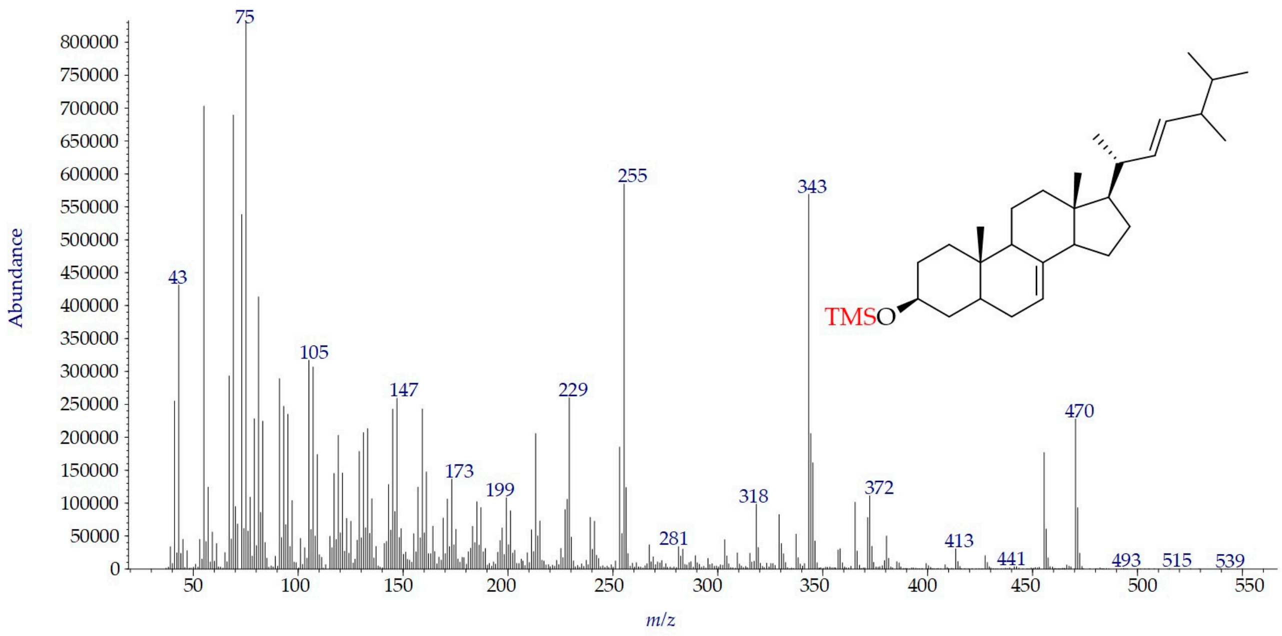

Figure S37. EI mass spectrum at 70 eV of ergosta-7,22-dien-3-ol (**15**), TMS (RI = 2589). TMS = trimethylsilyl group.
